# Potential of Flow Cytometric Approaches for Rapid Microbial Detection and Characterization in the Food Industry—A Review

**DOI:** 10.3390/foods10123112

**Published:** 2021-12-15

**Authors:** Elena Zand, Antje Froehling, Christoph Schoenher, Marija Zunabovic-Pichler, Oliver Schlueter, Henry Jaeger

**Affiliations:** 1 Department of Food Science and Technology, Institute of Food Technology, University of Natural Resources and Life Sciences Vienna (BOKU), 1190 Vienna, Austria; elena.zand@boku.ac.at; 2 Leibniz Institute for Agricultural Engineering and Bioeconomy, Quality and Safety of Food and Feed, 14469 Potsdam, Germany; afroehling@atb-potsdam.de (A.F.); oschlueter@atb-potsdam.de (O.S.); 3 Institute of Sanitary Engineering and Water Pollution Control, University of Natural Resources and Life Sciences, 1190 Vienna, Austria; christoph.schoenher@boku.ac.at (C.S.); marija.zunabovic@boku.ac.at (M.Z.-P.)

**Keywords:** flow cytometry, fluorescence in situ hybridization, inline monitoring, microbial contamination, food safety

## Abstract

As microbial contamination is persistent within the food and bioindustries and foodborne infections are still a significant cause of death, the detection, monitoring, and characterization of pathogens and spoilage microorganisms are of great importance. However, the current methods do not meet all relevant criteria. They either show (i) inadequate sensitivity, rapidity, and effectiveness; (ii) a high workload and time requirement; or (iii) difficulties in differentiating between viable and non-viable cells. Flow cytometry (FCM) represents an approach to overcome such limitations. Thus, this comprehensive literature review focuses on the potential of FCM and fluorescence in situ hybridization (FISH) for food and bioindustry applications. First, the principles of FCM and FISH and basic staining methods are discussed, and critical areas for microbial contamination, including abiotic and biotic surfaces, water, and air, are characterized. State-of-the-art non-specific FCM and specific FISH approaches are described, and their limitations are highlighted. One such limitation is the use of toxic and mutagenic fluorochromes and probes. Alternative staining and hybridization approaches are presented, along with other strategies to overcome the current challenges. Further research needs are outlined in order to make FCM and FISH even more suitable monitoring and detection tools for food quality and safety and environmental and clinical approaches.

## 1. Introduction

Microbial contamination, including the carryover of infectious microbes, is a global public health concern [1]. Food and beverage production and medical areas, including hospitals and pharmaceutical manufacturing, are of particular concern. Although clean rooms and sterile air filtration techniques are standard within medical areas, their use in the food industry is limited, mainly due to high construction and maintenance costs and the non-sterile conditions in production plants. Consequently, the increased persistence of microbes within food areas has been observed, even though hygiene standards have been massively increased nowadays with the implementation of HACCP, GMP, and GHP [2,3,4]. Microbial resistance against extrinsic factors is related to their fast adaptability and the formation of microbial biofilms, which can protect spoilage microorganisms and bacterial pathogens from chemical and physical actions [2,3,5,6]. Frequently detected pathogens include *Salmonella* spp., *Listeria monocytogenes*, *Escherichia coli*, *Shigella* spp., *Vibrio* spp., *Campylobacter jejuni*, and *Yersinia* spp., whereas frequent spoilage microorganisms are, for instance, *Acinetobacter* spp., *Pseudomonas* spp. or *Botrytis* spp. [7,8,9,10]. According to the WHO [11], 600 million foodborne infections in 2010, from which 420,000 people died, were associated with bacterial pathogens. Furthermore, the number of unreported foodborne diseases is outstandingly high [11].

Effective qualitative and quantitative monitoring and detection tools are required to minimize the contamination risk. The gold standard among detection tools is still the conventional plating method, with its high sensitivity and selectivity [12,13]. However, plating is time-consuming, labor-intensive, and detects only viable and cultivable microbes [7,14]. Complementarily, there are several rapid and culture-independent approaches that overcome these limitations of plating. Among the most widely-known detection methods are molecular methods such as polymerase chain reaction (PCR) or enzyme-linked immunosorbent assay (ELISA) methods [7,13,15,16]. Some molecular methods are vulnerable to interference from inhibitory compounds (i.e., the lipid content) or can affect complex matrices such as food. For highly sensitive methods such as PCR, contamination can easily lead to false results. In addition, PCR may be unable to distinguish between viable and non-viable cells [13,17].

An alternative technique that serves as a powerful, rapid, and highly sensitive [13,18] method for the specific and non-specific detection, monitoring, enumeration, and characterization of microorganisms is flow cytometry (FCM). FCM allows a culture-independent quantitative count of microbial cells. In addition, flow cytometry provides information on the physiological and structural characteristics of microbial cells and their viability and can therefore be used as an additional characterization tool. Rapid and reliable detection, quantification and characterization of foodborne pathogens are of great interest to the food industry in order to minimize foodborne diseases [19]. The rapid techniques used to detect foodborne pathogens can be categorized into immunological, biosensor, and nucleic acid-based methods [20]. Fluorescence in situ hybridization (FISH) is a nucleic acid-based method and is mainly applied in the medical and diagnostic field [19].

Even though current rapid detection approaches such as PCR or ELISA overcome the limitations of culture-based techniques, they do not meet all the criteria required, including effectiveness, reproducibility, rapidity, and sensitivity [13]. This review, therefore, provides detailed information on currently developed FCM and Flow-FISH protocols for the non-specific and specific detection, monitoring, and characterization of microbial contamination.

## 2. Microbial Habitats and Detection Targets within the Food Industry and Bioindustry

For the efficient and rapid detection of microbial contaminants, potential microbial habitats need to be considered in order to adapt detection tools such as FCM and the preceding sampling procedures. Generally, water, air and abiotic as well as biotic surfaces are parts of microbial contamination routes.

### 2.1. Water

Water is indispensable within the food and bioindustry for processing or washing steps and cleaning equipment and (non-) food contact surfaces. Wastewater can be either re-used or drained off [21,22]. Water monitoring was previously proposed as an alternative for pathogen detection within the food industry [23,24]. For example, drain water was proposed to be less biased than small-area swabs and is often linked to drain biofilms [24]. As a result, the drain water itself and the drain biofilm matrix are crucial for detecting microbial communities [22]. Any stagnant water, such as floor drains, can turn into a contamination source, further re-contaminating food or other products via spray water or aerosols [25].

### 2.2. Air and Aerosols

The air is a potential source and distributor of microbes within the processing areas [26]. Aerosols are often related to cleaning operations but can also originate from people, rotating equipment, or raw materials [4]. Air-borne contamination usually depends on the microbial load within the air and the exposure time between the product and the air, i.e., during sedimentation. In addition to sedimentation, air particles can also interact with the product or surface through cooling or heating procedures [26]. There are multiple approaches for reducing or preventing air-borne contamination, including appropriate air exchange rates and filtration technologies, maintaining positive pressure in critical areas, high pressure near doorways or other openings, and restraining airflow out of non-critical areas [4]. These ventilation-based tools are easily applied within clean rooms; however, their use within significant production areas is not always practicable.

### 2.3. Abiotic and Biotic Surfaces

Adherent microbes are found on all kinds of abiotic surfaces, including conveyor belts, stainless steel or polymer surfaces, gaskets, floors, and walls, as well as process units within the food and bioindustry, as well as medical devices [27,28,29]. These interactions between microbes and abiotic surfaces are accompanied mainly by biofilm growth [30,31] and the biofilm-forming ability is generally affected by the physicochemical and topographical properties of the respective food contact surfaces [32]. Other natural habitats for microbes are biotic surfaces, including fresh and/or raw food products such as meat, sprouted seeds, vegetables, or salad. For plant-based products, contamination can already take place during crop growth through the soil, water, or the use of fertilizers [21,33]. Most cross-contamination, however, occurs during process steps from a contact surface to the product, such as after the slicing of meat [5]. As the microbial safety of fresh food products is an emerging public health concern, alternative preventive methods for detecting pathogens and spoilage microorganisms, instead of end-product analysis, are required [34,35].

Due to the frequent occurrence of biofilms and their high relevance as hygiene and safety concerns, detection and avoidance are of the utmost importance. In this regard, rapid detection tools need to be applied to allow real-time process monitoring. For this purpose, innovative rapid and preventive control strategies are necessary. FCM and Flow-FISH are promising tools for online and inline detection and monitoring of microbial contamination from water, air, and abiotic (i.e., conveyor belts or pipelines) and biotic surfaces (i.e., solid food samples). The currently available FCM and Flow-FISH applications for detecting and monitoring water, air, and food matrices are discussed in the following two sections.

## 3. Non-Specific State-of-the-Art Flow Cytometric Applications for Detection and Monitoring

### 3.1. FCM Principle and Detection Mechanisms

**Figure 1:**
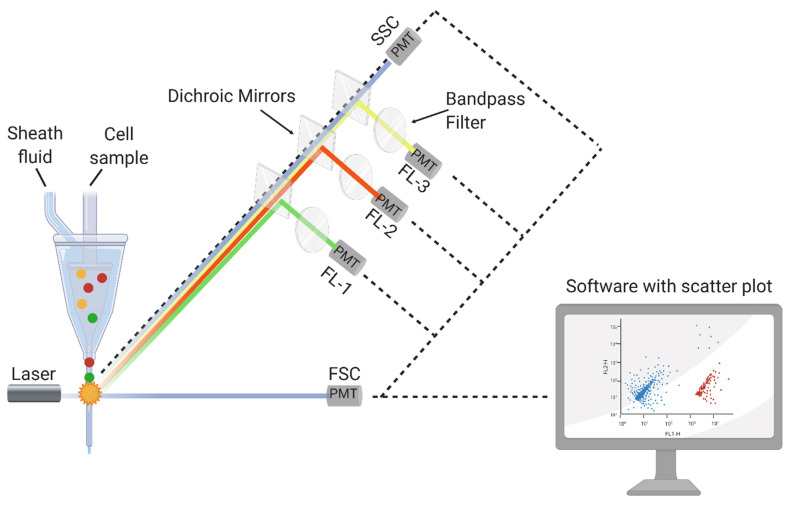
Principle of a flow cytometer (FCM). Forward-scattered light (FSC); side-scattered light (SSC); photomultiplier tubes (PMT, fluorescence detectors); detectors at a specific wavelength (FL-1, FL-2, and FL-3); (made in ©BioRender—biorender.com, Toronto, ON, Canada (accessed on 8 June 2021)).

In principle, FCM allows the analysis of the chemical and physical characteristics of any suspended single particle. The optical system of an FCM is illustrated in Figure 1. Usually, it contains the following: a flow chamber, a source of light (i.e., a laser or mercury lamp), dichroic mirrors to bring the light beam into focus, bandpass filters for the detection of different wavelengths, detectors (i.e., photodiodes (PD) and photomultiplier tubes (PMT)) for the detection and amplification of the signals, as well as a data processing unit [36,37,38,39]. After transferring the particles into a laminar flow of sheath fluid, scattered light and fluorescence signals are utilized one by one at the interrogation point of the laser beam. To differentiate cells regarding their morphology (i.e., particle size or granularity), forward- (FSC) or side-scattered light (SSC) is detected, respectively. Aside from scattered light, fluorescence appears when fluorochromes or particles labeled with them emit light, which is then excited by a beam of an appropriate wavelength. Some cells can emit fluorescence without fluorochromes, which is called autofluorescence. This phenomenon can be either beneficial for analysis or can impede other fluorescence signals. Most of the time, autofluorescence alone is not sufficient to detect and distinguish between cell populations. Thus, FCM protocols include a staining step with one or more fluorescent dyes before sample analysis [17,40].

Depending on the FCM protocol, various quantitative data can be obtained, including cell vitality, viability, or rather the physiological status and the stage of the growth cycle [18]. For the analysis of physiological status, fluorescent dyes, or rather fluorochromes, can target enzymatic activity, membrane integrity, pump activity, or membrane potential [41]. Dyes that intercalate with double-stranded nucleic acids are often used to assess the nucleic acid content, total cell count, or cell viability based on the cell membrane integrity [42]. For the latter, a combination of a permeant (i.e., Hoechst dyes, dyes from the SYTO- or SYBR-family, thiazole orange (TO)), and an impermeant dye (i.e., ethidium bromide (EtBr) or propidium iodide (PI)) is used. Moreover, the electrochemical membrane potential is another target for the testing of cell viability. Here, cationic dyes (i.e., 3,3′-Diethyloxacarbocyanine Iodide (DiOC_2_(3))) accumulate in polarized cells and anionic dyes (i.e., Bis-(1,3-Dibutylbarbituric Acid)Trimethine Oxonol or rhodamine 123 (DiBA-C_4_(3)) amass in depolarized cells, consequently emitting different fluorescent signals for viable and non-viable cells [43]. Changes in membrane composition can be measured by the extension of fluidity with 1,6-Diphenyl-1,3,5-hexatriene (DPH), indicating changes in the physiological state. Esterase activity or dehydrogenase activity, both being enzymatic mechanisms, are suitable for detecting, e.g., endospore viability or sublethally injured cells [44,45]. Respiratory activity is typically examined by loading cells with permeant nonfluorescent stains such as tetrazolium dyes, which are then converted to fluorescent substances by dehydrogenases. The most popular one is 5-cyano-2,3-ditolyl tetrazolium chloride (CTC), which is converted into the impermeant red molecule formazan [45]. For esterase activity assessments, fluorescin diacetate (FDA), carboxyfluorescein diacetate (cFDA), or acetoxymethyl ester (calcein-AM) are frequently used [46,47]. Another method for viability testing is the pump activity, in which the dye (i.e., ethidium bromide, rhodamine 123) loaded into the cells is again pumped out of the cells, increasing the fluorescent signal [48]. Moreover, changes in the intracellular pH can also be evaluated, with specific dyes that change their fluorescent signal depending on the pH value. Intracellular pH is a good target when assessing external effects on bacterial cells [46]. Furthermore, physical or structural parameters are also assessable. Gram staining, for instance, is rapidly performed with two fluorescent dyes, namely, hexidium iodine and oregon green, targeting Gram-negative and Gram-positive cells, respectively.

A quantitative FCM analysis can also provide qualitative data, such as specific cell clusters. Therefore, it can be further used to characterize microbial communities. FCM techniques, such as fingerprinting methods, can enhance the derivation of qualitative information [49]. With so-called fluorescence-activated cell sorters (FACS), single cells or subpopulations are sorted from a mixed population based on one or more specific characteristics and can be used for further analysis or growth [50]. Furthermore, specific targets such as food pathogens [51], infectious bacteria [52], and environmental contaminants are easily detected by FCM combined with phylogenetic labeling. Flow-FISH is a phylogenetic labeling method in which specific nucleic acid sequences inside intact viable cells are labeled [53], as further discussed in Section 4.

### 3.2. Food-Related FCM Applications

For food-related research, FCM is mainly used for the performance testing of food preservation or disinfection approaches, i.e., sodium hypochlorite or peracetic acid disinfection, ultraviolet light (UV-C), supercritical CO_2_ pasteurization, ohmic heating applications, non-thermal inactivation technologies, including pulsed electric fields and cold atmospheric pressure plasma treatment, as well as natural preservatives such as essential oils [47,54,55,56,57,58,59,60,61,62]. The most commonly investigated microorganism was *E. coli* [3,55,56,57,59,60,61,63,64,65,66].

A study by Coronel-Leon et al. [67] tested the antimicrobial effect of the surfactant N^α^-lauroyl L arginine ethylester monohydrochloride as a food additive and used FCM to understand the inactivation mechanisms better and to indicate the presence of sublethally injured cells. The most popular cell target for viability staining is membrane integrity, in which DNA-intercalating dyes are applied. Moreover, esterase activity is a suitable detection target, as potential sublethal injured cells after inefficient inactivation procedures are observed. For this purpose, FDA or cFDA are combined with PI [44,59]. Tamburini et al. [60] concluded that FCM was the most suitable viability assessment method compared to PCR, plate counts, and fluorescence microscopy.

FCM is not only a suitable tool for detecting sublethally injured cells but also for cells in the viable but non-culturable (VBNC) state. This is important as environmental stresses present during food processes, such as temperature change, pH, or the absence of nutrients, can introduce cells into the VBNC state. With culture-based techniques, only viable and culturable cells are detected. VBNC cells, however, are able to resuscitate and become culturable again [68]. Thus, FCM viability staining, in combination with plate count, can be conducted to detect the VBNC state of cells. Khan et al. [64] optimized staining protocols for VBNC enumeration by eliminating the interference with other particles and optimized the cell concentration to 10^4^ cells mL^−1^. Another study by Yu et al. [66] increased the sensitivity. It significantly reduced the background signals of impurity particles with the use of a high-sensitivity flow cytometer to detect microbes within the VBNC state. Comprehensive information about the role of sublethally injured cells and cells within the VBNC state on food safety matters is summarized in a review by Schottroff et al. [69].

Moreover, bacterial counts and viability in fermented products, including wine or probiotic products, were previously monitored with FCM. For wine samples, the viability and growth dynamics of yeasts and bacteria were measured, but prior washing steps had to be included to eliminate interference from other particles [70]. With the stain ChemChrome, viability assessment was possible even while natural particles were present in the sample [71]. According to Bunthof and Abee [72], FCM is highly sensitive and more accurate than plate counts.

Most of the available food-related studies use FCM for liquid samples, including juice, tea, water, wine, probiotic products, and milk, just to name a few. Fröhling, Durek, et al. [73] used FCM to evaluate indirect plasma treatment of fresh pork meat. Within their study, the meat samples were homogenized and centrifuged to remove the remaining meat debris (Table 1). For viability analysis of remaining bacterial cells after plasma treatment, cFDA and PI staining was successfully conducted. In another study, Juzwa et al. [3] applied a combination of FCM and cell sorting to improve microbial strain isolation from stainless steel surfaces within a fruit and vegetable processing company. Surfaces were swabbed with sterile cotton swabs and immediately resuspended in buffer solution for further FCM analysis.

### 3.3. Water and Bioaerosol FCM Applications

This section focuses on the characterization of drinking water quality and wastewater purification [74] and bioaerosol detection [49,75,76,77,78,79,80,81,82]. Although FCM has been widely used for the microbial analysis of aquatic milieus (Table 2), it is less frequently used for the quantitative detection of bioaerosols (Table 3) [80,83].

The determination of the total cell count (TCC) is probably the most straightforward FCM protocol available and requires only one nucleic acid binding stain. In 2012, TCC measurement via FCM was included in the guidelines for drinking water analysis in Switzerland [87]. The viability of live/dead analysis, with additional information on the intact cell count (ICC), can be supportive in analyzing the infectious risk, treatment efficiency, or inactivation process [88,89,90]. Here, the membrane integrity is targeted with a combination of cell-permeant and cell-impermeant nucleic acid stains. The most frequently used fluorochromes for this purpose are SYTO and SYBR stain families (cell-permeant dyes), i.e., SYBR^®^ Green I (SG1) or SYTO 9^TM^, together with propidium iodide (PI; cell-impermeant dye) [82,91]. Ma et al. [85] applied a rapid staining protocol with SG1-PI (green vs. red fluorescence) to quantify the TCC of bacteria and viruses, as well as the amount of live/dead bacterial cells during wastewater purification. An ultrasonication treatment was necessary to obtain free single cells for FCM analysis. With TCC measurement, even more complex datasets may be obtained, as fluorescence signals and scattered light create a so-called fingerprint of bacterial communities [92]. A study by Liang, Soupir, Rigby, Jarboe, and Zhang [84] distinguished environmental *E. coli* cells attached to clay or free from clay particles based on SSC gating.

Gating of SCC and green fluorescence allow the differentiation between bacteria with high and low nucleic acid contents [75,78,81,82]. Identifying and distinguishing between high- and low-nucleic-acid-content clusters can help to characterize water communities and are widely used for marine environments [81]. High-nucleic-acid-content cells were reported to be more sensitive and dynamic to changes, whereas low-nucleic-acid-content bacterial cells were associated with inactive or dead cells [82,93]. However, recent studies found out that bacteria containing low nucleic acid contents were metabolically active. A study by Prest et al. [81] linked drinking water contamination to increased high-nucleic-acid-content cells. It demonstrated a correlation between a rise in TCC and that of high nucleic acid content concentrations. A recent study by Farhat et al. [78] suggested that high-nucleic-acid-content bacteria showed increased sensitivity to chlorine dioxide, whereas low-nucleic-acid-content cells were more sensitive to ozone treatment.

In addition to high and low nucleic acid content, cytometric fingerprints allow for more holistic data analysis. A study by Favere et al. [49] implemented the Bray–Curtis dissimilarity as an online tool to differentiate between microbial communities in drinking water. This parameter is an easy unequivocal tool and was first developed by Bray and Curtis [94]. Two fingerprints are compared in regard to their dissimilarity and valued between zero (identical samples) and one (entirely different samples) [95]. FCM fingerprinting enables rapid monitoring and a sensitive early warning of changes or contamination in aquatic milieus and provides real-time monitoring and detection within 10 min as a fully automated online tool [49,75,92].

In contrast to water analysis, only a few publications have focused on the flow cytometrical detection of air-related microbes [77,80,86]. A study by Lange et al. [80] was the first to utilize FCM and FISH as a quantification and identification method for airborne microorganisms from agricultural surroundings and showed similar results to those obtained using fluorescence microscopy. For air sampling, an all-glass impinger-30 and a May multistage liquid impinger were used, comprising a collection liquid containing a surfactant (Tween 80) and an antifoaming agent (Antifoam A). Day et al. [77] differentiated air-borne *Phytophtora infestans* spores to pollen and other fungal spores by applying FSC, SSC, autofluorescent measurements, and the use of multiple gatings. Their study also demonstrated a more effective differentiation with the Calcofluor white M2R dye, which was characterized as non-toxic and showed a brighter fluorescence compared to other stains. One recent study coupled FCM and qPCR to quantify *Aspergillus versicolor* within indoor air [86]. A liquid cyclone air sampler was used for air sampling, and the particles were collected in sterile water. FCM was then used to rapidly count and calibrate *A. versicolor* particle concentrations before quantification with qPCR.

## 4. Specific State-of-the-Art Flow-FISH Methods and Applications for Monitoring and Detection

### 4.1. Principle of FISH

DeLong, Wickham, and Pace [96] were the first to describe FISH for microorganisms. The method based on the use of fluorescently-labeled oligonucleotide probes that target a specific region of rRNA (16S/23S in Bacteria/Archaea or 18S/28S in Eukarya) enables the specific identification of microorganisms from the domain to the subspecies level [96,97,98,99]. It is now a well-established technique [100]. In addition to oligonucleotide probes, fluorescently labeled antibodies can also be used for the identification of microbial cells, but the low cost of oligonucleotide probes and the availability of a large number of rRNA sequences, as well as the associated possibility of the in silico design of oligonucleotide probes, have led to the preferred use of oligonucleotide probes [101].

In contrast to culture-dependent methods, microorganisms that are difficult to cultivate can be identified. FISH visualizes whole cells, and since abundant structures in living cells are targeted, it is possible to distinguish between viable and dead cells, which is the main advantage over other molecular techniques such as PCR-based methods [1,98]. Additionally, the direct observation of cells within their native environment is possible [102,103]. Flow-FISH, a combination of FISH and flow cytometry, was described in the early 1990s by R.I. Amann et al. [104]. The advantage of Flow-FISH is that the method enables the rapid analysis of larger sample volumes, while being more convenient since manual counting is omitted [105].

**Figure 2:**
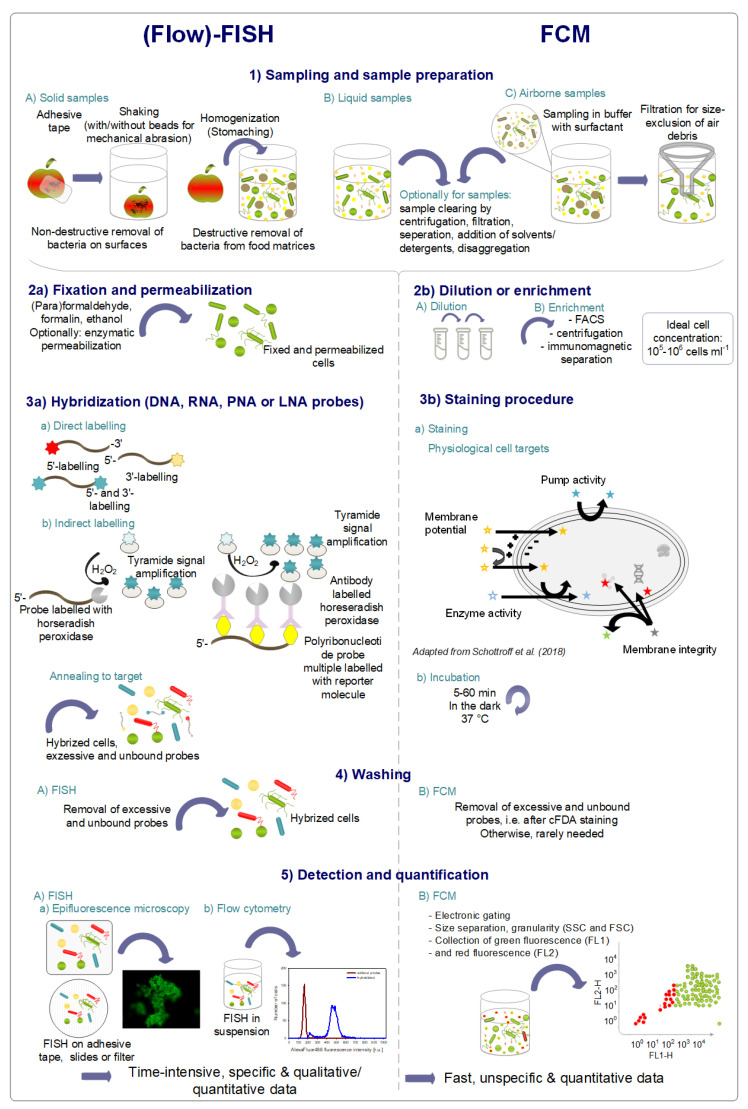
Standard sample preparation protocols and detection mechanisms of specific flow fluorescence in situ hybridization (FISH) and non-specific flow cytometry (FCM). LNA, locked nucleic acids; PNA, peptide nucleic acids.

In general, FISH consists of four preparation steps: (i) fixation and permeabilization, (ii) probe hybridization with the target sequence, (iii) washing of excess and unbound probes, and (iv) observation of cells with epifluorescence microscopic techniques, scanning microscopy, or flow cytometry (Flow-FISH) [98,100,106]. Sampling, pre-preparation, and hybridization steps, compared to a typical FCM protocol for quantitative analysis, are illustrated in Figure 2. The fixation and permeabilization procedure of samples has several purposes. Cells have to be fixed to stabilize cell morphology and so that they can withstand further processing, and microbial contamination and decomposition are prevented [97]. Furthermore, fixation protects the RNA molecules from degradation, and permeabilization enables the fluorescent probes to penetrate into the cells [107]. Commonly used fixation and permeabilization agents are (para)formaldehyde and ethanol [98], whereas 3%–4% (para)formaldehyde has been shown to be sufficient for Gram-negative bacteria, and for Gram-positive bacteria 50% ethanol, a mixture of ethanol and formalin (9:1), or heat treatment is suggested [107]. However, no standard fixation and permeabilization protocols are available since the cell wall composition of microorganisms differs, and modifications such as the addition of enzymes to digest peptidoglycan layers or proteinaceous cell walls, the addition of solvents to remove wax, or the addition of detergents are reported [101]. Following the fixation and permeabilization, oligonucleotide probes specifically bind to their target sequence in the hybridization step. Briefly, temperatures between 37 °C and 50 °C for 30 min to several hours in a dark, humid chamber are the conditions for hybridization [108]. Very stringent requirements for hybridization must be observed to ensure the specific binding of oligonucleotide probes to the target sequence. Additionally, the time and temperature needed for hybridization and the concentration of salts and denaturants have to be optimized for each application [98]. However, during the optimization of hybridization and washing conditions, it has to be considered that nonspecific hybridization at low temperatures and the loss of hybridized probes at high temperatures has to be avoided. The temperature affects the conformation of the targeted DNA or rRNA and, in addition to that, the accessibility of the target site to the probes. Moreover, the dissociation of the probe is affected by the temperature. Thus, the melting point of the probes, as well as the accessibility of the target site, determines the optimal hybridization and washing temperatures [108]. Formamide is commonly used in the hybridization buffer since it decreases the melting temperature by weakening the hydrogen bonds; thus, high stringency is achieved at lower hybridization temperatures [107]. Before the observation of the hybridized cells, a washing step is necessary to remove unbound and excessive probes to minimize false-positive detection [98]. Optionally, anti-fading agents are used to prevent the fluorescence from bleaching [107].

In general, DNA and RNA probes with a length of 15 to 30 nucleotides are used for FISH analyses with 16S rRNA as the predominant target sequence, but 23S rRNA is gaining more importance in research since it might enable differentiation between closely related strains due to the longer length of the probes. Fluorescent dyes used for FISH are typically fluorescein, tetramethylrhodamine, Texas red, and carbocyanine dyes such as Cy3 and Cy5 [1,100]. Alexa Fluor dyes and quantum dots (nanosized crystal particles) are among the new generation of fluorophores [109]. The probes can be labeled with fluorophores in different ways (Figure 2). Labeling the 5′-end with one or more dye molecules is achieved chemically during synthesis through an amino linker. Terminal transferases are used to enzymatically attach fluorescently-labeled nucleotides to the 3′-end of the probes. Labeling of both the 5′-end and 3′-end lead to an increase in the fluorescent signal. This direct labeling of oligonucleotide probes is the most commonly used technique since it is the easiest, fastest, and cheapest method [107]. Indirect labeling in terms of enzymatic signal amplification was developed to increase the sensitivity of FISH. Thereby, the oligonucleotide probes were labeled with horseradish peroxidase and fluorescein-tyramide as the substrate, leading to a 10–20-fold higher fluorescence signal due to signal amplification [110].

Another approach to increase the sensitivity of FISH analyses was the use of polyribonucleotide probes labeled with several dye molecules or the use of polyribonucleotide probes labeled with the reporter molecule digoxygenin combined with the tyramide signal amplification method [111,112]. Alternatives to oligonucleotide probes are so-called DNA mimics such as peptide nucleic acids (PNA) and locked nucleic acids (LNA) [109]. PNA is a synthesized DNA, having a comparatively high affinity to nucleic acids because the sugar-phosphate backbone (negative charge) is replaced with a pseudo-peptide [19]. Due to this non-charged polyamide backbone, PNA molecules are less susceptible to salt concentrations and repulsive forces, and therefore higher thermal stability between PNA and target is achieved. Other advantages of PNA are its ability to hybridize with nucleic acids at low salt concentrations and high temperatures, the stability of the molecule during storage, and its enhanced diffusion into the cells due to its nonpolar characterization with the ability to penetrate even into biofilm structures [1]. LNA is an RNA analogue in which the ribose ring between the 2′ oxygen and the 4′ carbon is locked by a methylene bond. This increases the local organization of the phosphate backbone and reduces the conformational flexibility of the ribose. The basic advantage of LNA is the possibility to speed up the hybridization process due to the possibility of increasing the melting temperature [19].

### 4.2. Flow-FISH in Food Microbiology

Flow-FISH in food microbiology is used to detect microorganisms in food products or for biofilm studies on abiotic surfaces, e.g., food contact surfaces. For food products, the range of food products examined is wide, from vegetables, meat, fish products, and dairy products to vinegar, wine, beer, and water. An overview is listed in Table 4.

FISH DNA or PNA probes are used for biofilm characterization, and the analyzed biofilms are either natural biofilms or those formed under laboratory conditions. FISH was used by Almeida, Azevedo, Santos, Keevil, and Vieira [158] to characterize and quantify the biofilm formation of *S. enterica*, *L. monocytogenes*, and *E. coli* on different surfaces (e.g., glass, stainless steel) using PNA probes. Stainless steel was also used as a surface to capture biofilm formation of various *Arcobacter* species using FISH in a study by Šilhová, Moťková, Šilha, and Vytřasová [161]. Bragança, Azevedo, Simões, Keevil, and Vieira [159] screened natural biofilms in a drinking water distribution system for the occurrence of *H. pylori* using PNA-FISH and evaluated the composition of natural biofilms from conveyors in breweries.

In general, the analysis of solid and liquid food products by means of flow-FISH often requires additional sample preparation steps (Figure 2) [162]. The presence of a high amount of proteins and fats might disturb the hybridization of the probes and these may have to be removed. Unspecific proteinases, dilution with appropriate buffers, homogenization, centrifugation, and filtration steps are applied to sample liquid food products. Different sample preparation steps must be performed depending on whether only microorganisms from the surface or from the entire product are to be detected. Homogenization with buffers or nutrient broth is followed by several filtrations and/or centrifugation steps to remove larger particles. In the case of surface sampling, the use of adhesive tape showed promising results. Additionally, pre-enrichment steps might be necessary for low numbers of target microorganisms in the food product. However, it has to be taken into account that the sample preparation steps might impact microorganism detection [1].

### 4.3. Flow-FISH in Water and Bioaerosols

In addition to food products and food contact surfaces, Flow-FISH is also applied to water (Table 5) samples and bioaerosols (Table 6). Water samples were either spiked with the relevant microorganism or the composition of the natural load was investigated. Additionally, water samples were analyzed in regard to the occurrence of specific microorganisms. FISH was applied to tap water samples to detect *E. coli* [163], *C. coli*, and *Mycobacterium avium* [164,165]. However, most studies dealing with FISH for water samples have focused on seawater and lake water samples. Here, FISH methods with tyramide signal amplification and without tyramide signal amplification have been applied [166,167,168,169,170,171,172,173,174].

For air-related applications, the natural load of bioaerosols in swine barns and buildings was successfully characterized using FISH [80,175,176]. Additionally, *Legionella pneumophila* in water aerosols were accurately detected using FISH [178]. Neef and Kämpfer [177] demonstrated the highly specific detection of microorganisms in bioaerosols from compost plant, performing the treatment within two working days using FISH, compared to weeks using cultivation techniques.

Even though FCM and flow-FISH have potential as innovative and fast monitoring and detection methods within the food and bioindustries, there are still food-related limitations.

## 5. General and Food-Related Limitations for Specific and Non-Specific Methods

### 5.1. Instrumental Limitations

According to a review by Wu et al. [39], challenges and limitations of bacterial analysis with FCM are mainly related to instrumental background noise or background from the sheath or sample fluid. With conventional FCM, it is thus difficult to distinguish small-sized bacteria (<0.5 µm) from background signals by means of light scattering alone. A study by Zacharias et al. [83] concluded that FCM worked well for monoculture but had difficulties with mixed populations. This leads to the assumption that the final interpretation of FCM data could be challenging for unknown microbial populations present in food products. It is also well-known that a specific cell concentration is required for optimal staining and cell counts. Cell aggregates in particular can lead to underestimated cell counts, as two or more particles might be considered one large particle by the electronic system [179]. This coincidence occurs more frequently at cell concentrations >2.5 × 10^6^ mL^−1^ (~1000–1400 events s^−1^). This can be reduced by using a low cell concentration or flow rate and through disaggregation via sonication [17,180]. According to a review by Wilkinson [40], most FCM protocols work best with 10^5^–10^6^ cells mL^−1^, whereas in a study by [181], 10^4^–10^8^ cells mL^−1^ showed optimal results for the live/dead analysis of a mixture bacteria population. They also argued that beyond these limits, false-positive readings were significant in association with interference from the sheath fluid or electronic system. In most of the studies summarized in Section 5 and Table 1 and Table 2, a cell concentration of 10^4^–10^7^ cells mL^−1^ was suggested. Therefore, samples either need to be concentrated or diluted before FCM analysis.

### 5.2. Hazardous and Toxic Substances for FCM and FISH Applications

Another remaining challenge is the use of hazardous and toxic fluorescent dyes, as they may pose health and safety risks. As discussed in Section 3.2 and Section 3.3, TCC or viability assessments are the most straightforward FCM assays, often performed with SYTO 9 or SYTO 9 and PI, respectively. Stiefel, Schmidt-Emrich, Maniura-Weber, and Ren [182] critically assessed the binding properties of those two dyes. In their study, it was concluded that SYTO 9 showed ineffective binding properties to intact Gram-negative cells, and also bleaching of SYTO 9 was reported. In comparison, PI staining can result in unwanted signals, also called background fluorescence, due to fluorescence in the unbound form. A study by Rosenberg, Azevedo, and Ivask [183] concluded that PI staining could underestimate the cell viability of adherent cells, as in biofilms, as extracellular nucleic acids outside intact cell membranes were stained red (PI), while at the same time, intracellular nucleic acids were stained green (SYTO 9).

Moreover, for a comprehensive viability assessment, the sole use of DNA-intercalating dyes (such as SG1 and PI or SYTO 9 and PI) is not sufficient, whereas assessments of esterase or respiratory activity are recommended in addition [59,83]. In contrast, a study by Kennedy et al. [53] further indicated that the detection of esterase activity and membrane potential, measured with a PI–calcein acetoxymethyl ester (calcein-AM) mix and DiOC_2_(3), respectively, could have resulted in false-positive events after physiochemical treatment of samples with heat and chemicals. These findings lead to the conclusion that the selection of fluorescent dyes has to be performed individually for each experiment.

Regarding safety, EtBr [184], propidium monoazide, PI [185], SG1 [186], lactofuchsin [187], and TO [188] are some examples of synthetic dyes that pose a health risk to humans and other organisms, as well as to the environment, after waste disposal. They were previously reported as being carcinogenic, toxic, or at a minimum, strongly allergenic. Furthermore, fluorescent dyes are also expensive.

For the hybridization step required for FISH methods, toxic and potential teratogens such as formamide are commonly used. As cell permeabilization is affected by the Gram characteristics of the microorganisms and also by the bacterial growth phase, while applying permeabilization treatments, cell integrity has to be maintained and cell loss lysis has to be avoided [106,162]. The specificity of the oligonucleotide probes determines the reliability and accuracy of the FISH method. Since the stringency of the applied protocol is directly correlated with the specificity of the probes, the stringency of the hybridization buffer and wash solution has to be considered to allow the proper annealing of probes to the target site. To adjust the stringency, formamide and sodium chloride are used, and in general high-stringency hybridization in combination with similar or lower-stringency washing is applied to obtain higher specificity [162]. However, for formamide, specific safety measures and precautionary steps are required.

Therefore, the necessity of the development of safer and more sustainable methods is arising [189]. Non-toxic, economic, and eco-friendly stains were previously discussed as a safer choice than the widely-used ones and these thus have future potential for viability or Gram staining [185,187,190,191]. The use of alternative substrates is further discussed as a new approach in Section 6.

### 5.3. Limitations of Traditional Flow-FISH Protocols

Specificity, sensitivity, and test speed, as well as economic aspects, play essential roles when considering the routine monitoring of food safety using flow-FISH [162]. Several challenges or limitations of FISH methods have to be taken into account. The first challenge is the food matrix itself. Due to strong background and autofluorescence signals, the food matrix is a critical influencing parameter for FISH methods, even though PCR-based methods are more biased by the matrix than FISH methods [162,192]. The detection limit of FISH methods is also a limitation. Unfiltered samples showed a detection limit of 10^5^ CFU/mL on slides, but 10^3^–10^4^ CFU/mL have also been given as detection limits. However, the detection limits reveal the necessity to include pre-enrichment steps to fulfill the requirements to be comparable to standard detection methods [1,98]. Pre-enrichment steps also overcome the weak fluorescence signals of classic rRNA-targeted FISH. Low-fluorescence signals are attributed to a low ribosome content in metabolically inactive or slowly growing cells in environmental samples or insufficient cell permeability to allow the penetration of the probes into the cells [106].

### 5.4. Interference with Food Matrices

In addition to the selection of appropriate fluorescent dyes, food matrix molecules, i.e., milk proteins [193], debris in wine [70], or any particles showing auto-fluorescence can interfere with the fluorescent dyes and impact the assay. Complex formulated food such as ready-to-eat meals offer additional challenges, as DNA-intercalating cell viability stains can non-specifically bind to, e.g., food flavorings containing DNA or RNA [194]. According to [71], probiotic drinks were suspended in buffer media and filtered (20 µm filter) to avoid biases due to food matrix particles. To exclude debris from wine samples, the work by Malacrinò et al. [70] used prior centrifugation, and additional washing steps. To eliminate proteins and lipids present in milk, the working group of Gunasekera et al. [178] treated their samples with either proteinase K or savinase. Bunthof [72] applied a milk-clearing solution, resulting in the flocculation and coalescence of milk micelles and the lysis of somatic cells. With this approach, they increased the particle size of interfering cells to make their removal easier.

### 5.5. Challenges for Rapid Bioaerosol Detection

There are additional challenges to overcome for the fast detection and monitoring of air-borne microbes within food and agricultural environments. Depending on the environment, air samples often have to be concentrated, but the recovery rate of concentrated samples via centrifugation or filtration has been reported to be highly variable [195]. As already mentioned in Section 3.3, Day et al. [77] tested the applicability of two flow cytometer systems for the differentiation of air-borne *P. infestans* spores to pollen and other fungal spores. Different evaluation strategies such as multiple gating, the use of FSC, SSC, and autofluorescence measurement need to be applied to distinguish between FCM histograms for pollen, fungal spores, and *P. infestans*. Possible reasons for the complexity of air-related measures include the high similarity of significant background events and, depending on the sampling location, low cell concentrations (i.e., 10^2^ CFU m^−3^ in the indoor air of homes) [80]. A clear and reliable distinction between background and bacterial events, as is already available for water analysis, must be accomplished [49]. Furthermore, the lack of published work on this topic [196,197] indicates that fast FCM detection for air-borne microbes has not yet been successful carried out. We believe that adapted sampling strategies that are compatible with FCM measurements are required, as well as specifically designed buffer solutions, and instrumental adaptations to overcome the high background signals. However, an adapted online air-sampling and FCM analysis chain could be of interest for the rapid detection of air-borne particles.

## 6. Strategies to Overcome Limitations and to Improve Detection Methods

### 6.1. Alternative Non-Hazardous Stains and Solvents to Improve Safety

Depending on the detection target and study aim, alternative fluorescent substrates could replace synthetic dyes to increase environmental and human safety and to lower costs. With alternative staining, this article refers to the use of substances that present one or more of the following characteristics: non-allergenic, non-toxic (also including noncytotoxic characteristics), noncarcinogenic, cost-effective, environmentally friendly, or biodegradable substrates. To give some applicable examples of alternative staining, a study by Vujanovic et al. [187] suggested fruit extracts for the cell wall staining of pathogenic *Fusarium* species via fluorescent microscopy. In their study, the highest fluorescence intensity was obtained by cranberry and currant extracts. Cell wall staining is typically used for the quantitative analysis of morphological or development studies but has also been proposed to have the potential for physiological research, as the cell wall structure adapts to physiological and environmental states [198]. It is also worth mentioning that Kurutos et al. [188] designed non-cytotoxic monomethine cyanine dyes for the microscopical fluorescent staining of chromosomes from human cell lines and could replace TO with this new staining approach. They found that the cyanine-based dye was 100× less toxic, showed a 5× higher photostability, and a higher (>800-fold) fluorescent intensity than TO. The present study will not further address staining methods for human cells, but aims to present the universal applicability of alternative staining methods that have been previously published. Outside of the field of fluorescence microscopy and flow cytometry, safer staining choices are becoming widely accepted in agarose gel staining and real-time PCR assays. Zhu et al. [199] reported the applicability of synthesized non-toxic and cost-effective copper nanoclusters for DNA staining, instead of EtBr, in gel electrophoresis. Furthermore, a study by Haines et al. [186] discussed the applicability of four commercially available replacements for SG1 and EtBr for agarose gel staining. Non-toxic and non-mutagenic characteristics were reported for the nucleic acid stains GelGreen^TM^ and RedSafe^TM^. Moreover, GelRed^TM^ and Diamond^TM^ Nucleic Acid Dye displayed weak mutagenic properties and toxic and mutagenic properties at stock concentrations, respectively. In contrast to this study, Sayas et al. [184] reported that both GelRed^TM^ and RedSafe^TM^ are toxic and mutagenic for *S. cerevisiae*, with RedSafe, however, being the safest dye. Furthermore, SYTOX Green, CellTox Green, and EvaGreen were previously found to be non-cytotoxic and cell impermeant when investigated with fluorescence microscopy [200]. According to a study by Tashyreva, Elster, and Billi [201], SYTOX Green was effectively used to visualize membrane-comprised cells of cyanobacteria when using fluorescent microscopy, with the minimal effective staining concentration ranging between 0.2 and 0.3 µm, and the significantly highest amount of stained cells was investigated after 90 min of incubation. Another innovative staining method for fluorescence microscopy was developed by Lin et al. [185], who designed carbon dots from *L. plantarum* LLC-605 exopolysaccharides, namely, CDs-EPS605, for live/dead analysis. The exopolysaccharides were purified, and quantum dots were fabricated via simple hydrothermal carbonization. The quantum dots were characterized as eco-friendly and cheap in production, and further showed negligible cytotoxicity, good fluorescence emission properties, as well as photostability. To conclude, a study by Kotenkova, Bataeva, Minaev, and Zaiko [202] recently assessed the potential of EvaGreen for flow cytometrical viability analysis of *L. monocytogenes* ATCC 13932. Their study reported signals in the green and red fluorescence spectra, indicating viable as well as dead cells with EvaGreen staining. However, as EvaGreen is cell-impermeant, DMSO was used to permeabilize the cell membrane, and thus the permeability of EvaGreen was influenced by the DMSO composition and the cell type used. Like EvaGreen, most of these non-toxic dyes, e.g., GelRed^TM^, GelGreen^TM^ [186], or SYTOX Green [201], are cell-impermeant and thus have potential as PI replacements. Their use instead of SG1 or EtBr replacements for flow cytometrical assays, however, requires an additional pre-treatment step, such as DMSO, to permeabilize the cell membranes. Interestingly, this was the only study found in relation to flow cytometry applications.

However, pretreatment steps are also required for flow-FISH protocols. Hybridization protocols require the use of the toxic solvent formamide for the reliable annealing of oligonucleotide probes to the target site, and studies are being conducted to replace formamide with non-toxic substances and still to achieve reliable annealing of the probes to the target sequence. Matthiesen and Hansen [203] revealed promising results using ethylene carbonate in non-toxic concentrations instead of formamide for tissue FISH, whereas Kalinka, My, and Achrem [204] and Golczyk [205] successfully substituted formamide with ethylene carbonate in plant FISH. Urea was used as a formamide alternative for the detection of gene expression in diverse animal species [206]. Aistleitner et al. [207] used urea as a formamide substitute for bacterial FISH and showed that the same specificity and similar signal intensities for the probes used in this study could be achieved after urea hybridization in comparison to hybridizations performed with formamide.

The lack of studies, however, underlines that this topic remains largely neglected for cytometry assays. We propose that alternative staining and hybridization procedures are crucial to maintaining safety for food and bioindustry applications in the long run.

### 6.2. PEF-Assisted FCM and Flow-FISH Approaches

The use of non-toxic alternative substrates, shorter incubation times, and increased fluorescence intensity signals are required to optimize FCM as a monitoring and detection tool for the food industry and the bioindustry. The permeabilization of cells could help to improve these three criteria, as impermeant (staining) molecules can immediately pass the cell wall; thus, no incubation time with the staining solution is needed, and fluorescence intensity will be increased if more staining molecules can enter the cells. For cell permeabilization, well-known pre-treatment steps include ethanol fixation, RNase digestion, and enzymatic cell wall digestion with, for instance, pepsin, proteinase K, or snailase treatment. Zhang and others (2018) compared three different methods for yeast cell permeabilization, including snailase digestion, DMSO, and electroporation, as pretreatments for yeast DNA staining. They suggested that the use of DMSO (50% (*v*/*v*) for 20 min at 60 °C) is the best method to improve PI staining in different basidiomycetous yeasts, as it is quick, simple, and cheap. In their study, however, they did not show any data observed using electroporation pre-treatment. However, DMSO is also discussed to show at least low mutagenicity [208] and toxicity [209]. An electroporation-based pretreatment with pulsed electric fields (PEFs) might be a safe, as well as a quick and cost-efficient, alternative. Electroporation of the microbial cell membrane is induced by generating high-intensity electric fields (kV cm^−1^) for repetitive short time periods (µs). The formation of pores can either be reversible or irreversible, depending on the interplay of various conditions such as treatment intensity, electrical conductivity, and electrode configuration [62]. Permanent pores can result in membrane integrity loss and uncontrolled molecule transfer through microbial cell membranes [210]. Currently, many researchers are focusing on the use of PEF to permeabilize microbial cell membranes of, for instance, microalgae [211], yeast [212], and bacteria cells [213] to extract valuable substances. A recent review by Martínez, Delso, Álvarez, and Raso [214] summarized the advantages of PEF in comparison to chemical and physical cell permeabilization or disruption methods. They argued that PEF is energetically efficient, non-destructive, highly selective, easy to scale up, as well as low in operational costs, has no thermal effects, and requires only a short processing time. Furthermore, PEF showed inactivation effects in different food matrices, such as in milk [58,215] and fruit [216], and vegetable juices [217] are typically analyzed using FCM. Although there are many studies on PEF-assisted extraction and FCM characterization of PEF-induced damage, there are only a few studies within the food sector on the use of electroporation for channeling molecules into cells [218]. However, loading cells with small molecules is commonly used within the medical area, e.g., in electrochemotherapy or gene electrotransfer [219,220]. This PEF-assisted FCM detection method could be used to load microbial cells with a non-toxic nucleic acid stain for TCC analysis, to be used as a first indicator of microbial contamination. However, all these applications (extracting molecules, loading cells with molecules, and the inactivation of cells) are based on the permeabilization of the cell membrane by means of PEFs. For some applications, i.e., for cultivation after FCM analysis or for cell sorting, reversible electroporation is essential to maintain the metabolically active state of the cells. This is, however, already challenging with single-strain populations in suspensions, as a population contains microbes within different growth states and the homogeneity of PEF treatment is still challenging. A study by Vaessen, Timmermans, Tempelaars, Schutyser, and Den Besten [221] demonstrated two different staining protocols—first with PI only, and second with a double-staining method prior to and after PEF treatment using PI and SYTOX Green—to analyze PEF permeabilized *L. plantarum* cells. With the double staining method, they were able to find a larger reversible permeabilized fraction, with 40%. Their study demonstrates that the reversible permeabilization of microbial cells is still challenging, as up to 100% of the cells should be targeted to obtain representative data about quantitative microbial cell counts. Another study by Zand, Schottroff, Schoenher, et al. [222] suggested a rapid single-staining method with SYTOX Green and additional gating to describe intermediate cellular states of *E. coli*, such as sublethal effects, induced by PEF.

Moreover, the improved permeabilization of cell membranes is of interest for alternative staining methods and during FISH to allow the penetration of probes into the cells. To date, permeabilization has been realized enzymatically or chemically, but there is always the risk of cell lysis [106]. Additionally, concerning a possible fast, automated detection method applied in routine (online) food analysis, chemical substances are undesirable in a production plant, as they could contaminate the products. Furthermore, commonly used dehydration steps, which lead to an increase in the probe uptake, are unsuitable for rapid automatic detection due to the need for elaborate sample preparation. Fröhling, Nettmann, Jäger, Knorr, and Schlüter [223] showed that the use of PEFs to permeabilize *E. coli* cells increased the hybridization rate, and a reliable detection using flow cytometry was possible. Electroporation was also successfully used by Ruark-Seward, Davis, and Sit [224] for the FISH analysis of nematodes, and a reduction in time and specimen losses, and increased hybridization efficiency was achieved, showing the potential of electroporation for cell permeabilization in FISH methods. These successful FISH-related applications lead to the assumption that PEF pre-treatment will potentially also benefit alternative staining methods for quantitative FCM approaches.

### 6.3. Optimized Flow-FISH Concepts

Various modifications of classical FISH procedures are described in the literature. Volpi and Bridger [225] assembled an excellent overview of FISH techniques. Since the classical FISH methods often result in low fluorescence signals, several improved FISH methods are available to overcome these drawbacks. Double labeling of oligonucleotide probes (DOPE)-FISH is based on the use of double-labeled probes (at the 5′ and 3′ ends), which doubles the fluorescence signals and enables the simultaneous detection of six microbial populations [207,226]. Using catalyzed reporter deposition (CARD)-FISH, a 26- to 41-fold-higher sensitivity than that of standard FISH is achieved [110,227]. Additionally, it was shown that CARD-FISH could detect mRNAs of virulence factors in *L. monocytogenes* [112]. However, it has to be considered that the enzyme-labeled probes require the higher permeability of the cell membranes [227,228]. The two-step hybridization chain reaction (HCR)-FISH is a radical-free alternative to CARD-FISH, which is performed without formaldehyde fixation, with the advantage that DNA quality and genome amplification are not impaired. HCR-FISH was used in combination with FACS to enrich bacteria for genomic analysis [229]. Improvements of FISH using RNase-H-assisted rolling circle amplification enabled the visualization of mRNA expression at the single-cell level and, combined with meta-analysis, allowed researchers to gain insights into the role of individual microorganisms in the microbiota [230]. Batani, Bayer, Böge, Hentschel, and Thomas [231] developed a Live-FISH protocol in combination with FACS, which allowed the isolation and culturing of targeted bacteria. It is suggested that the method might have the potential to enhance the culturing of new microorganisms from diverse environments.

### 6.4. Improvements of FCM-Based Bioaerosol Detection

According to the reviews of Chen and Li [196] and Yoo et al. [197], FCM in combination with fluorescent techniques is a potential tool for the fast quantitative detection of air-borne microbes (~1000 cells s^−1^), apart from qPCR or bioaerosol mass spectrometry, but it is rarely used for this purpose. The following recent findings underline the potential of innovative FCM strategies for bioaerosol quantification. Choi, Kang, and Jung [232] designed an integrated microchip flow cytometer, a so-called micro-optofluidic platform, for the real-time detection and quantification of air-borne cells, and coupled this with a BioSampler^®^, a liquid-type particle collection setup, for the continuous sampling of airborne particles. Compared to microscopical investigations and colony counts, they were able to quantify the overall particle concentration within the air. They could distinguish *E. coli*, *B. subtilis,* and *S. epidermidis* bioaerosols from abiotic air debris when stained with SYTO82. Chang, Ting, and Horng [233] tested and compared three different liquid-based sampling techniques, including the cyclonic-based Coriolis^®^ µ Air Sampler, the BioStage^®^ Single-Stage Impactor, and the BioSampler^®^, in an animal farm, as well as a public library. Within that study, the BioSampler^®^ showed the highest efficiency in collecting fungal aerosols, as assessed based on colony counts. There is no literature combining the Coriolis^®^ µ Air Sampler or the BioStage^®^ Sampler with FCM to quantify air-borne microbes. In another work, Choi, Hong, Kim, and Jung [234] developed a liquid-based continuous microfluidic sampling device, the MicroSampler, for the real-time size-selective sampling of bioaerosols. The MicroSampler even showed good recovery of airborne *S. epidermidis* cells compared to conventional bioaerosol sampling techniques, such as the BioSampler^®^ technique. The integration of a liquid-based sampler into an optimized FCM detection technique could have potential for future bioaerosol characterization within the food and bioindustries.

## 7. Conclusions and Future Research Needs

To summarize, it can be stated that FCM has been successfully used to detect and monitor microorganisms in water samples and was included in the Swiss guidelines for drinking water analysis in 2012 (SLMB, 2012). Moreover, FCM and flow-FISH are promising tools for the detection, monitoring, and characterization of microbes within the food industry and the bioindustry. Even though methodical improvements and innovative techniques are available and have demonstrated the potential of FISH for the detection of food pathogens and the potential of FCM for physiological targets, FCM and FISH are not routinely used to analyze and monitor microbial contamination of liquid and solid food products. Furthermore, the combination of FISH and flow cytometry (flow-FISH) has not gained considerable interest in relation to the detection of microorganisms in food production areas to date. State-of-the-art FCM protocols still show significant limitations, including (1) time-consuming or complex sample preparations and/or staining procedures; (2) the use of toxic substances that pose a risk for human health as well as their carry-over into the system; (3) instrumental drawbacks; (4) their limits of detection, especially for FISH assays; and (5) biases due to interferences with food matrices. Moreover, (6) challenges related to the analysis of airborne particles need to be tackled (6). Aside from these aspects, it has to be stated that the equipment’s price and accessibility for food and bioprocessing labs is still limited. Future studies should focus on potential optimization strategies for FCM and FISH protocols, as well as on the development of automated lab-on-chip solutions.

To allow for rapid online and inline detection and monitoring within the food industry, non-hazardous alternative fluorochromes could be applied instead of toxic ones to avoid contamination of the analytical and production environment and risks for human health and safety. For the use of nontoxic fluorochromes, however, proper sample pretreatment steps must be implemented in order to maintain the performance of the analysis. PEF shows high potential as a pretreatment method, as it is non-destructive, no residues are generated, and has only low operational costs, as well as a short process time. It should be noted that the possibility of reversible permeabilization is of great importance for some applications in order to leave the cell physiology unaffected. PEF is not suitable as a pre-treatment step, for instance, if the effects of cleaning and disinfection on microbial cells are to be monitored, as PEF affects cell physiology. In contrast, for FISH, irreversible electroporation has been successfully applied as a permeabilization tool to increase the hybridization rate and speed up the analysis. Nevertheless, future research is necessary to overcome the heterogeneity of PEF and to validate the proposed PEF-assisted FCM detection approach. Combining FCM-based analysis with liquid-based sampling tools would also allow the detection and monitoring of airborne particles, as well as liquid and solid matrices.

## Figures and Tables

**Table 1:** State of the art FCM applications in liquid and solid food matrices, as well as abiotic surfaces.

Research Area/ Food Matrix	Model Microorganism/ Sample Type/ Sample Location	Detection Target, Fluorochrome(s), and Gating	Sample Preparation and Observation Methods	References
Wine	Yeasts (*Saccharomyces cerevisiae* and *Saccharomyces bayanus* strains) Malolactic bacteria (*Oenococcus oeni* strains); Fresh wine samples from different wineries	Viability (Rhodamine 123, calcein acetoxymethyl ester, 2″, 7″-bis(carboxyethyl)-5(6)-carboxyfluorescein acetoxymethyl ester, fluorescein diacetate (FDA))	Samples were diluted, centrifuged and suspended in PBS Incubation (5 min for yeasts and 15 min for malolactic bacteria cells) FCM and CFU enumeration	[70]
Milk, fermentation starters and probiotic products	*Lactiplantibacillus plantarum* WCFS 1 for milk samples Commercially available diary starters (mixed cultures) Probiotic products (mixed cultures)	TCC (SYTO 9) Viability, based on enzymatic activity and membrane-permeabilized cells (carboxyfluorescein diacetate (cFDA) and TOTO-1)	Milk samples: *L. plantarum* samples within the exponential growth phase were resuspended in semi-skimmed pasteurized milk and cleared The cheese starter was analyzed directly and after incubation Yogurt starters were analyzed without any further preparation Probiotic products (Yakult, Orthiflorplus, Mona Vifit yogurt drink) were cleared before sampling Total assay time (1 h) FCM compared to CFU enumeration and fluorescence microscopy	[72]
Non-dairy probiotic drinks and pharmaceutical products	Pure cultures: four different *Lactobacillus* strains and *Bifidobacterium animalis* subsp. *lactis* Two commercially available pharmaceutical probiotic products and six probiotic drinks	Intracellular enzymatic reaction and intact cell membrane (ChemChrome, cFDA, cFDA-AM, sFDA, and CAM) Viability (SYTO 9 and propidium iodide (PI))	Samples were suspended in either ringer solution or PBS Incubation with ChemChrome (10 min), with other esterase activity dyes (15–60 min) FCM, fluorescence microscopy and fluorometer	[71]
Pulsed electric field (PEF) inactivation Milk	*Lactobacillus rhamnosus* E522	Esterase activity and membrane integrity (cFDA and PI)	PEF-treated samples were centrifuged (2600× *g*, 10 min), resuspension in 50 mM PBS Incubation (10 min, 37 °C, cFDA), washing to remove excessive cFDA, incubation (10 min, on ice, PI) FCM and colony count	[58]
Detection of VBNC for increased microbiological safety	*P. aeruginosa* *Pseudomonas syringae* *S*. Typhimurium *E. coli* O157:H7	TCC, viability, and VBNC (SYTO 9, SYTO 13, SYTO 17, or SYTO 40 in combination with PI)	Strains were used at the late log phase in either King’s broth or Luria–Bertani broth (and heat-treated at 72 °C for 5–15 min) Total assay time (70 min), incubation (60 min) FCM and CFU enumeration	[64])
Disinfection efficiency and Mastitis detection Milk	*E. coli* DSM 1116	Cell membrane integrity (Thiazole orange (TO) and PI) Metabolic activity and membrane integrity (cFDA + PI) Cell membrane potential (3,3′-diethyloxacarbocyanine iodide (DiOC_2_(3))	*E. coli* was analyzed at stationary phase in PBS (pH 7.0) Incubation (TO + PI, 10 min; cFDA + PI, 45 min; DiOC_2_(3), 15 min) FCM, coulter counter and CFU enumeration	[57]
Food preservation	*E. coli* ATCC 11229 *L. innocua* ATCC 33090 *S. cerevisiae* KE162	Membrane integrity (PI) and esterase activity (fluorescein diacetate (FDA))	Strains were analyzed at the stationary phase in PBS buffer (pH 7.0) Incubation (PI, 10 min; FDA, 30 min) FCM and CFU enumeration	[59]
Indirect plasma treatment Fresh pork	Fresh pork (directly from the slaughterhouse)	Viability based on esterase activity and membrane integrity (cFDA and PI)	After plasma treatment of meat samples Homogenization of meat samples and centrifugation (200× *g*, 4 °C, 2 min) to remove meat particles Centrifugation of supernatant (4000× *g*, 4 °C, 6 min) Re-suspension (in 0.05 M PBS) Incubation (15 min, 37 C) FCM, fluorescence spectrometer, UV/Vis/NIR spectrophotometer, and colony count	[73]
Non-thermal plasma treatment Bacterial model system on polysaccharide gels	*L. innocua* DSM 20649 *E. coli* DSM 1116	Esterase activity (cFDA) Membrane integrity and RNA and DNA damage (TO and PI)	Gelrite^®^ polysaccharide gels were inoculated with 25 μL bacteria suspension After plasma treatment, bacteria were resuspended in 0.05 M PBS and agitated (5 min, 750 rpm, 37 °C) Incubation (15 min + 10 min for *L. innocua* and 45 min + 10 min for *E. coli*, cFDA and PI; 10 min, TO + PI) After cFDA and PI staining, centrifugation (4000× *g*, 6 min, 4 °C) to remove cFDA FCM and colony count	[56]
Fresh food preservation and analytical viability methods	*L. monocytogenes* *E. coli* *Salmonella enterica*	Viability SYBR^®^ Green I (SG1) and PI)	In vitro experiment: type cultures were incubated until stationary phase (16 h) Incubation (15 min) FCM vs. propidium monoazide quantitative PCR Reference methods: CFU enumeration and fluorescence microscopy	[60]
Drinking water and tea	*E. coli* ER2738	TCC, viability and VBNC state (PicoGreen, for tea samples: +1 mM EDTA ^2^) Gating with FSC and SSC	*E. coli* cells were used in stationary phase and spiked to either water or jasmine green tea sample Assay time (<20 min) New FCM approach and CFU enumeration	[66]
New inactivation technologies (peracetic acid, ozonated water, cold atmospheric pressure plasma) Fruits and vegetables	*E. coli* DSM 1116 *L. innocua* DSM 20649 *Pectobacterium carotovorum* spp. *carotovorum* DSM 30168	Membrane integrity and RNA/DNA damage (TO and PI) Esterase activity (cFDA) Membrane potential (DiOC_2_(3))	Treated samples were centrifuged (3220× *g* for 15 min, 4 °C) and resuspended in 50 mM PBS or directly resuspended in PBS and agitated (5 min, 750 rpm, 37 °C) Incubation (10 min, TO + PI; 15 min, DiOC_2_(3); 15 or 45 min for Gram-positive or Gram-negative bacteria, respectively, cFDA) FCM and colony count	[63]
Antimicrobial surfactant and food safety	*Yersinia enterocolitica* ATCC 9610 *L. plantarum* ATCC 8014	Viability (PI and bis-oxonol) The cell population was selected via gating of FSC vs. SSC Aggregates and cell debris were excluded	After treatment, strains were diluted in filtered buffered peptone water and stains were added Incubation (n.a. ^1^) FCM and transmission electron microscopy	[67]
Juice preservation	*S. cerevisiae* KE 162	Viability, esterase activity, and membrane integrity (FDA and PI)	*S. cerevisiae* cells were analyzed in either peptone water or carrot–orange juice Incubation (30 min, FDA; 10 min, PI) FCM, TEM and CFU enumeration	[44]
Essential oils against foodborne pathogens	*L. monocytogenes* Scott A *E. coli* MG 1655	Membrane integrity (TO and PI) Cell membrane potential (DiOC_2_(3)) Viability based on cell membrane integrity and esterase activity (cFDA and PI)	After treatment, bacterial cells were centrifuged (7000× *g*, 4 °C, 15 min), resuspended in 50 mM PBS, and centrifuged (7000× *g*, 4 °C, 5 min) Incubation (10–15 min, TO; 5 min, PI; 45 min, cFDA, and PI; 15 min, DiOC_2_(3)) FCM and colony count	[55]
Food-borne pathogens	*Staphylococcus aureus*	Viability (SYTO 9 and PI Cyanide 3-chlorophenylhydrazone (CCCP) and DiOC_2_ Calcein-AM, PI and cetyltrimethyl ammonium bromide (CTAB))	Cultures were cultivated in nutrient broth until exponential phase Incubation (30 min, calcein-AM; 15 min, other dyes) FCM and scanning electron microscopy	[53]
Lettuce disinfection	*E. coli* CECT 434	Viability (SYTO-BC and PI)	Inoculated and disinfected lettuce samples were suspended and stirred in 0.9% NaCl, from what 2 mL were removed for FCM and 200 µL for CFU enumeration Incubation (10 min) FCM and CFU enumeration	[61]
Microbial egg safety	Eggs spiked with pathogenic *Salmonella* Typhimurium and harmless *E. coli* K12	TCC	*E. coli* strain, clay, PBS, and fluorochromes were mixed at fixed volumes to a sample mixture Total assay time (1.5 h) FCM and settling method	[65]
Ohmic heating Paraprobiotics production	*Lactobacillus acidophilus* LA-5, *Lacticaseibacillus casei* 01 and *Bifidobacterium animalis* subsp. lactis Bb-12	Viability (TO and PI)	Ohmic heating treated samples were centrifuged (3500× *g* × 3 min, 4 °C), washed and resuspended in PBS Incubation (10 min, TO; 5 min; PI) FCM, SEM, plate counts, Gram staining, catalase test	[54]
PEF treatment Model solution for liquid foods	Model solution containing *E. coli* ATCC 9637	Viability (SG1PI)	PEF treated *E. coli* suspension (~10^5^ cells/mL) was immediately stored on ice until staining Incubation (13 min, SG1PI) FCM and plate counts	[62]
Microbial food safety—Contamination monitoring of stainless steel surfaces	Sampling location: Stainless steel conveyor belts after cleaning procedures Reference strains: *E. coli* ATCC 10536 *Sarcina lutea* *Bacillus subtilis* ATCC 6633 *S. aureus* ATCC 33592	Cellular redox potential and cell sorting for the identification and discrimination between active and non-active sub-populations (BacLightTM RedoxSensor^TM^ Green Vitality Kit, including FITC-A and PE-Texas Red-A) Viability (fluorescein isothiocyanate (FITC) and PI)	Swabs of 100 cm^2^ stainless steel areas within a fruit and vegetable processing company were taken and immediately placed in 2 mL 1% PBS solution In situ analysis Incubation (n.a. ^1^) FCM, cell sorting, and CFU enumeration	[3]

^1^ n.a., no information was provided in the publication; ^2^ EDTA, ethylenediaminetetraacetic acid; FCM, flow cytometry; PEF, pulsed electric fields; TCC, total cell count.

**Table 2:** State-of-the-art FCM applications for water monitoring.

Research Area/Analyzed Matrix	Model Microorganism/ Sample Type/ Sample Location	Detection Target, Fluorochrome(s), and Gating	Sample Preparation and Observation Methods	References
Microbial particle transition from environmental to water samples	*E. coli* (three environmental strains; one modified pathogenic strain)	Viability (SYTO 11) and propidium iodide (PI) and VBNC state Cell distinction is mainly based on SSC scattering, as unattached *E. coli* cells show low SSC and attached cells indicate high SSC	*E. coli* strain, clay, PBS, and fluorochromes were suspended at defined volumes Total assay time (~1 h) FCM vs. settling method	[84]
Aquatic milieu/water	*Legionella pneumophila* and *E. coli*	LIVE/DEAD^®^ BacLight^TM^ Bacterial Viability Kit (SYTO 9 and PI)	Cells were harvested at an exponential growth phase and heat-treated according to the experimental plan prior to FCM analysis Incubation (15 min, Syto 9, 25 min, Syto 9 and PI) FCM vs. propidium monoazide quantitative PCR	[83]
Drinking water	Bacterial cells within the native drinking water	TCC and permeabilized membranes (SYBR^®^ Green I (SG1) and PI) Gating to distinguish between high- and low-nucleic-acid-content bacteria	Collected at a drinking water tap of a distribution system Samples were buffered with 10 mM borate (pH 8.0) Incubation (14–18 min) FCM	[82]
Drinking water	Drinking water samples	TCC (SG1) and distinction between high and low nucleic content Fixed gating between green and red fluorescence was used, whereas for low and high nucleic acid content gates were set on the green fluorescent spectra	n.a. ^1^ Total assay time (<15 min), incubation (10 min) FCM	[81]
Drinking water	Groundwater site in Switzerland (further used for drinking water)	TCC (SG1) Gating to distinguish between high- and low-nucleic-acid-content bacteria	Sampling was conducted every 15 min during 14 days Incubation (10 min) Online FCM with automated staining module	[75]
Drinking water	Samples from drinking water treatment plant	TCC (SG1) Gating to distinguish between high- and low-nucleic-acid-content bacteria	Sampling every 10 min for 10 days Total assay time (10–30 min), incubation (10 min) FCM and heterotrophic plate count	[78]
Drinking water	Sampling location: incoming and existing water streams of water towers	TCC (SG1) Bray–Curtis dissimilarity ^2^ to assess dissimilarities between microbial communities	Automated online sampling every 40 min from all streams Real-time monitoring, incubation (20 min) Online FCM	[49]
Drinking water Disinfection	Fungal spore suspensions (*Asperigillus niger*, *Trichoderma harzianum*, and *Penicillium polonicum*)	Viability (SG1 and PI) Esterase activity (cFDA) Measurement of ROS levels (dihydroethidium, DHE)	EDTA was added to the spore solution (10^5^–10^6^ cells/mL) Incubation (10 min, cFDA; 20 min; DHE; 25 min, SG1PI) FCM and plate counts	[76]
Wastewater monitoring	Different wastewater samples (bacteria and viruses)	Total bacterial count and live/dead (SG1 + PI) Total viral count (SG1 + EDTA)	Samples were collected from a wastewater treatment plant (in northern China) Total assay time (45 min), Incubation (10 min, viruses; 25 min, bacteria) FCM, ATP, and epifluorescence microscopy	[85]

^1^ n.a., no information was provided in the publication. ^2^ The Bray–Curtis dissimilarity is derived from cytometric fingerprints. It quantifies the difference between two cytometric fingerprints. EDTA, ethylenediaminetetraacetic acid; FCM, flow cytometry; FISH, fluorescent in-situ hybridization; SSC, side scatter; TCC, total cell count; qPCR, quantitative PCR; VBNC, viable but nonculturable state.

**Table 3:** State-of-the-art FCM applications for bioaerosol detection.

Research Area/Analyzed Matrix	Model Microorganism/ Sample Type/ Sample Location	Detection Target, Fluorochrome(s), and Gating	Sample Preparation and Observation Methods	References
Bacterial quantification in the air of an agricultural environment (swine confinement building)	*E. coli*	A distinction of bacterial cells from other debris (4′,6-diamidino-2-phenylindole (DAPI))	Aerosol collection with an all-glass impinger-30 and a May multistage liquid impinge Collection liquid (1% peptone, reverse-osmosis-purified H_2_O with 0.01% Tween 80 and 0.005% Antifoam A) Total sampling time: 30 min, flow rate: 12.5 L/min Incubation (overnight) FCM, fluorescence microscopy, colony count and FISH	[80]
Spore analysis and differentiation to other particles in air samples	*Phytophthora infestans* spores *Botrytis cinerea* and *Alternaria alternata* (isolated from potato tissues)	Spore staining [1,1′,3,3,3′,3′-hexamethylindodicarbo-cyanine iodide (DiIC_1_(5)), 3,3′-dipropylthiadicarbo-cyanine iodide (DiSC_3_(5)), TO-PRO-3 iodide, SYTO dyes (SYTO 17, 59, 60, 61, 62, 63 and 64), Nile Blue A, Calcocluor white M2R]	Suspensions were either filtered through a single layer of muslin or washed twice Incubation (15 min in the dark) FCM	[77]
Microbial contamination in indoor air	*Aspergillus versicolor*	FCM: Quantitative cell counts and calibration (with gating of FSC and SSC) qPCR: SYBR^®^ Green amplifications with Takara master mix	Bioaerosols were collected from 38 mold-damaged homes with a liquid cyclone air sampler (Coriolis, Bertin Technologies) FCM analysis time (200 s), incubation (n.a. ^1^) Real-time qPCR calibrated with FCM	[86]

^1^ n.a., no information was provided in the publication.

**Table 4:** FISH applications for microorganism detection in food matrices and abiotic surfaces.

Sample	Target Microorganisms	Target Probe (5′-3′ Sequence) and Fluorophore	Sample Preparation/Fixation/Observation Method	References
Tomato; Jalapeno; Cilantro; Spinach	*Salmonella* spp. (spiked)	*23S*: Sal3/Salm-63 cocktail *Fluorophore:* fluorescein; TexasRed, Cy5	Bacterial removal: adhesive tapes Liquid phase enrichment: tape + TSB or BPW (5 h, 37 °C) Fixation: pelleted (5 min, 2000× *g*), 10% formalin (30 min, 25 °C) Fluorescence microscopy; flow cytometry	[113]
Olive	*L. plantarum* (spiked/natural)	*16S:* LbpV3 (CCGTCAATACCTGAACAG) *Fluorophore:* fluorescein	Bacterial removal: olives in Ringer’s solution (overnight, RT, shaking); pelleted (8000 rpm, 5 min, RT); Ringer’s solution; Fixation: 4% paraformaldehyde Fluorescence microscopy	[114]
Sprouts	*S.* Typhimurium (spiked)	*23S:* Sal-3 (AATCACTTCACCTACGTG) *23S:* Salm-63 (GCTGCCTCCCGTAGGAGT) *Fluorophore*: Cy5; 6-FAM	Bacterial removal: sprouts + 0.1% PW homogenized (1 min, 230 rpm); vacuum filtered (4 layers of sterile cheesecloth; centrifuged (300× *g*, 30 s); Fixation: supernatant 10% formalin (1:2) 30 min Fluorescence microscopy; Flow cytometry	[115]
Swine carcasses	*Salmonella* spp. (natural)	*23S:* Sal3 (AATCACTTCACCTACGTG) *Fluorophore:* fluorescein	Bacterial removal: swab + BPW w + 0.1% Tween 80; homogenized (90 s) Pre-enrichment: 37 °C, 18 h Fixation: centrifugation (14,000 rpm, 10 min), washed twice (PBS); 4% paraformaldehyde (2 h) Fluorescence microscopy	[116]
Swine carcasses	*Salmonella* spp. (natural)	*23S:* Sal3 (AATCACTTCACCTACGTG) *Fluorophore:* fluorescein	Bacterial removal: swab + BPW w + 0.1% Tween 80; homogenized (90 s) Pre-enrichment: 37 °C, 18 h Fixation: centrifugation (19,500 rpm, 10 min), washed twice (PBS); 4% paraformaldehyde (4 h) Fluorescence microscopy	[117]
Minced pork meat	*Yersinia* spp. (spiked)	*16S:* YersEcoI16 (TATTAAGTTATTGGCCTTCCTCCT) *16S:* YersEcoII16 (TTAACCTTTATGCCTTCCTCCTC) *23S:* YersEco23 (CAAGTCCCTTTACCTAATGCCAGC) *23S:* YersPseu23 (ATCACGCCTCAGGGTTGATAAG) *16S:* YersPseu16 (GCGTATTAAACTCAACCCCTTCC) *23S, LNA:* YersPest1523-TexasRed (CTGCACCGTGGTGCATCGTC) *23S, LNA:* YersPseu1523-Alexa488 (CTGCACCGTAGTGCATCGTC) *16S:* Yersall-Demaneche (GTTCGCTTCACTTTGTATCT) *16S:* EUB-338 (GCTGCCTCCCGTAGGAGT) *Fluorophore:* Alexa488, TexasRed	Selective enrichment: PSB and ITC broth (48 h) Fixation: pelleted (14,000× *g*); 4% PBS/formaldehyde (2 h, 4 °C) Fluorescence microscopy	[118]
Pork sausage	*S. enterica* (spiked/natural)	*23S:* Sal-3 (AATCACTTCACCTACGTG) *Fluorophore:* fluorescein isothiocyanate (FITC)	Pre-enrichment: nutrient broth (12 h, 37 °C) Fixation: pelleted (12,500 rpm, 3 min); 4% paraformaldehyde (1 h, 4 °C) Fluorescence microscopy	[119]
Ground beef; Ground pork; Milk; Lettuce; Cooked shrimp	*L. monocytogenes* (spiked)	*PNA:* LmPNA1253 (GACCCTTTGTACTAT) *Fluorophore:* Alexa Fluor 568	Pre pre-enrichment: One Broth Listeria (24 h); 1:10 dilution in One Broth Listeria (18 h) Fixation: Smears on slides; 4% paraformaldehyde (10 min); 50% ethanol (10 min); air dry Fluorescence microscopy	[120]
Ground beef	*Clostridium perfringens* (spiked)	*16S:* CLP-180 (AATGATGATGCCATCTTTCAACA) *Fluorophore:* carboxytetramethylrhodamine (TAMRA)	Bacterial removal: sample + 0.1% peptone water (30 s homogenized) FISH in combination with filter cultivation: 0.1 mL food homogenate + 4 mL TSC-broth; filtered (hydrophilic polypropylene membrane filters); incubation (37 °C, 6 h) Fixation: 2 mL of ethanol (99.5%) (RT, 15 min) Fluorescence microscopy	[121]
Chicken	*C. jejuni* (spiked/natural)	*16S:* CAM 1 probe *Fluorophore*: 5(6)-carboxyfluorescein-N-hydroxysuccinimide ester (FLUOS); tetramethylrhodamine-5-isothyocyanate (TRITC)	Bacterial removal: irradiated product + nutrient broth (2 min homogenized); incubation (1 h at 37 °C) Pre-enrichment: pre-enrichment broth + Preston selective broth (microaerophilic, 37 °C, 22 h Fixation: pelleted; 4% paraformaldehyde (2 h, 4 °C) Fluorescence microscopy	[122]
Chicken breast	*C. jejuni* (spiked)	*23S:* Campy268 (AACCCCCAGTGCAAGCACTGGGTTTG)*23S, LNA:* CampyLNA268 (CCCCCAGTGCAAGCACTGGGTTT) *23S:* Campy696 (CACTAGTTCTTACACTAGCTTCAACTTGC) *23S:* Campy835 (CTACCCCCTTATATTACGACACAACGC) *23S*: Campy1508 (AGCCTTTCAGTTCTCGGAGT) *23S:* Campy2124 (CTGGCGTCATATACTCAAAGCCTC) *23S:* Campytherm (CTTAGCCCTAAGCGTCCTT) *Fluorophore:* Alexa488, Texas Red, AMCA	Pre-enrichment: 1:10 in Bolton broth, (1 min homogenized); incubation (microaerophilic 4 h; 37 °C + 44 h, 42 °C) Fixation: 4% formaldehyde (15 min); washed two times in water and end-fixed in 95% ethanol (5 min) Fluorescence microscopy	[10]
Minced lamb meat	*Salmonella* Enteritidis (spiked)	*23S*: Salm63 (TCGACTGACTTCAGCTCC) *Fluorophore:* Cy3	Pre-enrichment: BPW (37 °C, 18 h) Fixation: 4% paraformaldehyde (30 min, refrigerated) Fluorescence microscopy	[123]
Chicken scraps and gizzards; Beef; Pork; Bacon; Salami; Sausage; Fish; Egg; Milk; Milk powder; Cheese; Butter; Ice cream; Pudding; Bell pepper; Lettuce; Bean sprouts	*Salmonella* Panama (spiked); *Salmonella* spp. (natural)	*23S:* Sal-1 (ACAGCACATGCGCTTTTGTG) *23S:* Sal-3 (AATCACTTCACCTACGTG) *23S:* Sal-544 (GCAGTCACACAGGTAAAC) *Fluorophore:* Cy3	Pre-enrichment: BPW (up to 24 h, 37 °C) Fixation: pelleted (13,000 rpm, 2 min); 3.7% formaldehyde (4 °C, 1 h) Fluorescence microscopy	[124]
Fermented sausages; Cured ham; Turkey meat; Lamb meat; Chicken meat; Minced meat (pork and beef); Beef meat; Cottage cheese; Semi-hard cheese; Fresh cheese	Natural microbial community	*16S:* EUB338 (GCTGCCTCCCGTAGGAGT) *16S:* ALF968 (GGTAAGGTTCTGCGCGTT) *23S:* Bet42a (GCCTTCCCACTTCGTTT) *23S:* Gam42a (GCCTTCCCACATCGTTT) *23S:* HGC69a (TATAGTTACCACCGCCGT) *16S:* LGC354ab (YGGAAGATTCCCTACTGC) *16S:* Pae (TCTGGAAAGTTCTCAGCA) *16S:* Sth (CATGCCTTCGCTTACGCT) *18S:* EUK (ACCAGACTTGCCCTCC) *Fluorophore*:Cy3, 6-carboxyfluorescein (6-FAM)	Bacterial removal meat: PBS (1:4); mixed (5 min); filtered; pelleted (8000 rpm, 10 min) Bacterial removal cheese: PBS (1:4); mixed (5 min); two times centrifugation (buffer (100 mM Na_2_HPO_4_, 150 mM NaCl, 10 mM EDTA, 40 mM NaOH); 8000 rpm, 2 min) Fixation: ethanol/PBS (1:1) Fluorescence microscopy	[125]
Smoked salmon; Camembert; Uncured ham	*Listeria* spp. (spiked)	*23S:* Lis-1400 (CGCACATTTCCATTCGTGCGATTCC) *Fluorophore*: TAMRA	Bacterial removal: sample + 0.85% NaCl (30 s homogenized) FISH in combination with filter cultivation: 0.1 mL food homogenate + 4 mL TSC-broth; filtered (hydrophilic polypropylene membrane filters); incubation (37 °C, 12 h) Fixation: ethanol (30 min) Fluorescence microscopy	[126]
Smoked salmon; Mozzarella; Julienne cabbage	*L. monocytogenes* (spiked)	*16S:* mRL-2 (AGAATAGTTTTATGGGATTAGCTCCACC) *Fluorophore:* Alexa647	Bacterial removal: sample + 0.85% NaCl (30 s homogenized) FISH in combination with filter cultivation: 0.1 mL food homogenate + 4 mL TSC-broth; filtered (hydrophilic polypropylene membrane filters); incubation (37 °C,12 h) Fixation: 50% ethanol (1 h) Fluorescence microscopy	[127]
Ikura (traditional Japanese seafood); Minced chicken meat	*E. coli* (spiked)	*16S:* Enterobacteriaceae (TGCTCTCGCGAGGTCGCTTCTCTT) *Fluorophore:* TAMRA	Bacterial removal: sample + 0.8% NaCl (2 min homogenized) FISH in combination with filter cultivation: vacuum filtered through Isopore membrane filter (0.4 µm pore size); filter incubation (6 h at 37 °C, TSB) Fixation: ethanol (RT) Fluorescence microscopy	[128]
Zebra mussels	*Cryptosporidium parvum*, *Giardia lamblia, Encephalitozoon intestinalis, Encephalitozoon hellem, Enterocytozoon bieneusi* (natural)	Cry-1 (CGGTTATCCATGTAAAAG) Giar-4 (CGGCGGGGGGCCAATTAC) Giar-6 (CGGGGCTGCCGCGGCGCG) HEL878F (ACTCTCACACTAACTTCAG) INT-1 (GTTCTCCTGCCCGCTTCAG) BIEN-1 (AUCAACGAAUGACUUGA) *Fluorophore:* HEX, 6-FAM, Tet	Bacterial removal: mussel flesh was homogenized with sterile PBS; the homogenate was sieved, sedimented, and purified over a CsCl_2_ gradient Fluorescence microscopy	[129]
Stilton cheese	Bacteria (natural)	*16S:* S-D-Bact-0338-a-A-18 *Fluorophore:* fluorescein	Fixation: cheese (0.5 × 0.3 × 1 cm) + 3.7% formaldehyde in PBS (3 h); washed in 6.8% sucrose solution in PBS (overnight), dehydrated in acetone (1 h), infiltrated with a plastic solution (8 h); mixed with hardener II (5 min), covered with cover foil (4 °C, 5 h); 5 µm sections were cut (cryostat, 4 °C), immediately straightened in sterile water, attached to lysine-coated slides, air-dried Fluorescence microscopy	[130]
Livarot cheese	Bacteria, Yeasts, *Candida catenulata, Candida intermedia, Geotrichum* spp., *Yarrowia lipolytica* (natural)	*16S:* EUB338 (GCTGCCTCCCGTAGGAGT) *23S:* Gam42a (GCCTTCCCACATCGTTT) *23S:* HGC69a (TATAGTTACCACCGCCGT) *18S:* EUK516 (ACCAGACTTGCCCTCC) *26S:* Ccat (TTTATCTCCCGCGCCT) *26S:* Cint (TTATCCACCCCTAGCA) *26S:* Geo (TTACGGGGCTGTCACCCT) *26S:* Ylip (CACTCATTTCCTTCCC) *Fluorophore:* FITC, Cy3, rhodamine	Bacterial removal: cheese rind + 10 mL (2%) trisodiumcitrate (homogenized: 8000 rpm, 1 min); pelleted and resuspended in 1× PBS Fixation: ice-cold 4% paraformaldehyde (4 °C, 4 h) Fluorescence microscopy	[131]
Gruyère cheese	Bacteria; Actinobacteria, *Brevibacterium* (natural load)	*16S:* EUB338 (all bacteria) *16S:* HGC1901 (Actinobacteria) *16S:* BRE1239 (Brevibacteria) *Fluorophore:* Cy3	Bacterial removal: 10 cm^2^ of the surface + 0.8% NaCl/0.1% peptone solution (homogenized: 2 min) Fixation: pelleted (7000× *g*, 5 min); 4% paraformaldehyde/PBS (4 °C, 12 h) Fluorescence microscopy	[132]
Gruyère cheese	*Propionibacterium freudenreichii* (natural)	*16S:* Pfr435 (CTTGCGCTTCGTCATGGATGAAAG) *Fluorophore:* 6-FAM	Bacterial removal: 10 g cheese + 2% trisodium citrate (homogenized: 2 min), repeatedly filtered with sterile gauzes; cells were washed four times with sterile PBS and resuspended in 1/10 of the original volume; Fixation: cold 4% paraformaldehyde (4 °C, 16 h) Fluorescence microscopy	[133]
Italian cheese	*Enterococcus italicus* (natural)	*16S:* ESA452 (CATTCTCTTCTCATCCTT) *Fluorophore:* Cy3	Bacterial removal: cheese + sterile 2% sodium citrate solution (homogenized); centrifuged (8000× *g*, 15 min, 4 °C) repeated washings (3–5 times) with same buffer, pellets were dissolved in PBS Fixation: freshly prepared cold paraformaldehyde solution (4% in PBS) (4 °C, 16 h) Fluorescence microscopy	[134]
Egg; Milk; Mayonnaise	*S.* Enteritidis (spiked)	*PNA* (details not specified) *Fluorophore*: AlexaFluor 594	Pre-enrichment: sample + pre-warmed BPW (18–21 h, 37 °C) Fixation: 4% paraformaldehyde Fluorescence microscopy	[135]
Ground beef; Unpasteurized milk	*E. coli* O157 (spiked)	*23S, PNA:* EcoPNA1169 (CAACACACAGTGTC) *Fluorophore:* AlexaFluor 594	Pre-enrichment: sample + pre-warmed BPW or TSB+ novobiocin (18–21 h, 37 °C or 41 °C) Fixation: 4% paraformaldehyde; pretreatment with 1% Triton X-100 to remove background fluorescence Fluorescence microscopy	[136]
Raw milk; Pasteurized milk; Raw meat; Ready-to-eat meat; Seafood	*Listeria* spp. (spiked)	Lis-16S-1 (ACTGTTGTTAGAGAAG) Lm-16S-2 (TAGTACAAAGGGTCG) Lm-16S-3 (CGAATGATAAAGTGT) Lm-16S-4 (CGCATGCCACGCTTT) Liv-16S-5 (ACGCATGTCATCACT) *Fluorophore:* FAM	Enrichment: according to standard procedures Fixation: centrifugation (2000× *g*, 5 min), washed PBS, resuspended in PBS, 10 µL placed onto a microscope coverslip, air-dried, fixed with 80% ethanol (15 min) Fluorescence microscopy	[137]
Powdered infant formula	*Salmonella* spp. (spiked)	*23S, DNA:* Sal-3 (AATCACTTCACCTACGTG) *23S, DNA:* Salm-63 (GCTGCCTCCCGTAGGAGT) *PNA:* Sal23S10 (TAAGCCGGGATGGC) *PNA:* SalPNA1873 (AGGAGCTTCGCTTGC) *Fluorophore:* AlexaFluor 594	Pre-enrichment: sterile distilled water (8 h, 37 °C) Fixation: pelleted (10,000× *g*, 5 min), 4% paraformaldehyde (1 h) Fluorescence microscopy	[138]
Natural whey starters for Parmigiano Reggiano	*Lactobacillus helveticus, Streptococcus thermophilus* (natural)	*23S*: Lbh1 *23S:* St4 *Fluorophore:* FITC, Cy3	Bacterial removal: whey samples washed twice in PBS, pellets resuspended in PBS. Fixation: 4% freshly prepared cold paraformaldehyde (1 h, 4 °C) Fluorescence microscopy	[139]
Dairy starter cultures (PROBAT-like cultures)	*Lactococcus lactis* subsp. *cremoris, L. lactis* subsp. *lactis*, *Leuconostoc* spp. (natural)	*16S:* CREM62 (CCAATCTTCATCGCTCAA) *16S:* LAC62 (CCAACCTTCAGCGCTCAA) *16S:* LEUC1026 (CACTTTGTCTCCGAAGAG) *Fluorophore:* Oregon Green	Fixation: pure cultures and cleared PROBAT cultures resuspended in PBS; mixed with equal volume ethanol (96%) Flow cytometry; fluorescence microscopy	[140]
Dairy starter cultures	*Leuconostoc* spp. (natural)	*16S:* Leugen2 (GGGCATTACAAACTCCC) *16S:* CHCC2114 (ACTTCGTATCATGCGAC) *Fluorophore:* Cy3, FITC	Fixation: starter cultures centrifuged (5 min, 14,000× *g*) and washed in PBS; one volume of cell suspension was mixed with three volumes of freshly prepared cold paraformaldehyde solution (4% in PBS; 4 C, 16 h) Fluorescence microscopy	[141]
Milk	Enterobacteriaceae; *Pseudomonas* spp. (spiked)	*16S:* Enterobacteriaceae (TGCTCTCGCGAGGTCGCTTCTCTT) *16S:* Pseudomonas spp. (GATCCGGACTACGATCGGTTT) *Fluorophore:* FITC, Cy5	Milk clearing: 0.5 μL savinase + 100 μL 0.1% Triton X-100 + 100 μL milk (30 °C, 30 min) + 800 μL 150 mM NaCl solution, centrifuged (13,500× *g*, 5 min, 20 °C), resuspended with PBS; sample filtered (0.2 μm pore size) Colony formation: filter on Standard Methods Agar (37 °C (Enterobacteriaceae) or 30 °C (Pseudomonas spp.), 3–5 h) Fixation: microcolonies dehydrated by a filter paper soaked with 80% ethanol (10 min, RT), air-dried on a new filter paper. Fluorescence microscopy	[142]
Raw bovine milk	*Helicobacter pylori* (natural)	*16S.* Hpy-1 (CACACCTGACTGACTATCCCG) *Fluorophore*: TAMRA	Removal of particulate milk components: milk mixed 2% sodium citrate solution (1 min, speed setting “6” in a BagMixer), centrifuged (5000× *g*, 10 min, 4 °C), pellets washed twice with 2% sodium citrate; resuspended in PBS Fixation: 4% paraformaldehyde solution (4 °C, 16 h) Fluorescence microscopy	[143]
Ultra-heat-treated milk	*Bacillus cereus* spores (spiked)	*16S*: pB394 (ATGCGGTTCAAAATGTTATCCGG *Fluorophore:* Alexa488	Fat removal: 25% sodium citrate + milk (5 min, 200 rpm), centrifuged (15,000× *g*, 5 min), cream adhered to the wall removed; pellet resuspended in TSB + L-alanine + inosine (1 h, 37 °C, 150 rpm) Fixation: pelleted (12,000 rpm, 2 min), 4% paraformaldehyde (15 min) Fluorescence microscopy	[144]
Milk	*Pseudomonas* spp. (spiked)	*16S: Pseudomonas* spp. (GATCCGGACTACGATCGGTTT) *Fluorophore:* TexasRed (TR), FITC	Milk protein and fat removal: 10 µL savinase + 100 µL milk (30 °C, 30–45 min) + 0.15 M NaCl, centrifuged (10,000× *g*, 22 °C, 5 min), digested proteins and top layer drawn off; bacterial pellet suspended in hybridization buffer Flow cytometry; fluorescence microscopy	[145]
Milk	*Lactobacillus* spp. (spiked)	*16S, PNA:* Lac663 (ACATGGAGTTCCACT) *Fluorophore:* AlexaFluor488	Fixation: milk pelleted (10,000× *g*, 5 min), 4% paraformaldehyde (1 h) Fluorescence microscopy	[146]
Skimmed milk	*Propionibacterium freudenreichii, Lactococcus lactis* (spiked)	*16S:* GLO62 (AAGGGCCTTACCGTCCGA) *16S:* PEU64 (CAAGGGGCCTTACCGTCC) *16S:* PFX311 (GGCACGTTCCTCACGTGT) *16S:* LactV5 (GCTCCCTACATCTAGCAC) *Fluorophore:* Cy3, Cy5	Fixation: sample centrifuged (12,000× *g*, 2 min); pellets washed twice with PBS; pellet fixed in solution of PBS/ice cold ethanol (12 h, 4 °C) Fluorescence microscopy	[147]
Milk	*Pseudomonas* spp. (spiked)	*16S: P. putida* probe (TTGCCAGTTTTGGATGCAGT) 16S: *Pseudomonas* spp. probe (GATCCGGACTACGATCGGTTT) *Fluorophore:* Cy5	Fixation: CTC-stained cells in paraformaldehyde (final concentration: 8%, 4 °C, 1 h) Fluorescence microscopy	[148]
Wine	*Dekkera bruxellensis* (natural)	*PNA:* BRE26S14 *Fluorophore:* 5(6)-carboxyfluorescein	Pre-enrichment: wine samples filtered (0.45 µm pore size, HVLP filter membranes; incubation (BSM, 30 °C); grown colonies used without fixation Fluorescence microscopy	[149]
Wine	Lactic acid bacteria (natural)	*16S:* Lbrev (CATTCAACGGAAGCTCGTTC) *16S:* Lcoll (CTTGATTTAACGGGATG) *16S:* Lcory (GCTTCGGTCGACGTCAGT) *16S:* Lfarc (AGCTTCAATCTTCAGGAT) *16S:* Lhilg (CAACTTCATTGACCAAGACGCG) *16S.* Lmali (AAGCATTCGRTGAAAGTTTTG) *16S*: Lpara (GTTCCATGTTGAATCTCGG) *16S:* Lzeae(5′-TTCATCGACCAAAACTC-3′) *16S:* Ooeni (5′-TAGTCATTGCCTCACTTCACCCGAA-3′) *16S:* Pdamn (5′-GTTGAAATCATCTTCGA-3′) *16S:* Pparv (5′-CTAAAATCATCTTCGGTGCAAGCAC-3′) *Fluorophore:* rhodamine 6G, 5(6)-carboxy-fluorescein-N-hydroxysuccinimide ester, 6-FAM, Alexa Fluor 350, 5(6)-carboxy-tetramethylrhodamine-N-hydroxysuccinimide ester	Bacterial removal: wine samples filtered using a vacuum of 6250 mbar on black polycarbonate filters (0.2 µm pore size) Fixation: overlaying the filter twice with PBS; 96% ethanol added (3 min, RT); cell permeabilization with lysozyme Fluorescence microscopy	[150]
White and red must industrial fermentations	Yeasts (natural)	*26S:* Cst (CTCTATGGCGTTTCTTTC) *26S:* Hgu (CAATCCCAGCTAGCAGTAT) *26S:* Huv (TCAATCCCGGCTAACAGTA) 26S: Kma (AGCTACAAAGTCGCCTTC) *26S:* Kth (ATAGGACTAGACTCCTCG) *26S:* Pan (GACAGGCAATATCAGCAGA) *26S:* Pme (AGAGCTTCGCACGGCACC) *26S:* Sce (TGACTTACGTCGCAGTCC) *26S:* Tde (GCAGTATTTCTACAGGAT) *Fluorophore:* FITC	Colony preparation: yeast counting on CRB medium; 30 colonies picked for yeast identification Fixation: 4% paraformaldehyde (4 h, 4 °C) Fluorescence microscopy	[151]
Wine fermentation	*S. cerevisiae, Hanseniaspora guilliermondii* (spiked)	*26S: S. cerevisiae* (CAATCCCAGCTAGCAGTAT) *26S: H. guilliermondii* (TGACTTACGTCGCAGTCC) *Fluorophore:* FITC	Bacterial removal: samples centrifuged (5 min, 5000× *g*), cells washed once with PBS Fixation: 4% paraformaldehyde (3 h, 4 °C, strong agitation) Flow cytometry; fluorescence microscopy	[152]
Wine fermentations	*S. cerevisiae, Hanseniaspora uvarum, Starmerella bacillaris* (spiked/natural)	*26S:* Cst (CTCTATGGCGTTTCTTTC) *26S:* Hgu (CAATCCCAGCTAGCAGTAT) *26S:* Huv (TCAATCCCGGCTAACAGTA) *26S:* H8a (TGAGAGGCCCAAGCCCAC) *26S:* H8b (AGGTAATCCCAGTTGGTT) *26S:* H8b-Com (AGGCAATCCCGGTTGGTT) *26S:* Sce (TGACTTACGTCGCAGTCC) *26S:* Sba (CTCCATGGCGCTCCTTTC) *Fluorophore:* FITC	Bacterial removal: samples centrifuged (10,000 rpm, 5 min), resuspended in 1× PBS Fixation: 4% paraformaldehyde (1 h, 4 °C, 1000 rpm) Flow cytometry; fluorescence microscopy	[153]
Wine fermentations	*S. cerevisiae, H. guilliermondii* (spiked)	*26S: S. cerevisiae* (CAATCCCAGCTAGCAGTAT) *26S: H. guilliermondii* (TGACTTACGTCGCAGTCC) *Fluorophore:* FITC	Bacterial removal: samples centrifuged (5000 rpm, 5 min), resuspended in 1× PBS Fixation: 4% of paraformaldehyde for (4 h, 4 °C, 1000 rpm); DAPI/PI staining prior to fixation for viability testing Flow cytometry; fluorescence microscopy	[154]
Red and white wine	*Dekkera bruxellensis* (spiked)	*26S:* Dkb271 (CCTTCCTCCTCTCTAGT) *Fluorophore:* ATTO 647N	Bacterial removal: cells recovered via centrifugation, washed with PBS Fixation: absolute ethanol (1 h, RT) Flow cytometry; fluorescence microscopy	[155]
Beer	*P. cerevisiiphilus, Pectinatus frisingensis* (spiked)	*16S:* Pf469 (CATTCACTATACTTATTGGC) *16S:* Pc469 (CATTCAATAGTGGTATTAAC), *16S:* Pc593 (AAGATCCGCTTAGTCATCCG), *16S:* Pc640 (AAGATGACCAGTTCGAATCC) *Fluorophore:* FITC	Bacterial removal: centrifugation (5 min, 10,000× *g*), suspended in sterile PBS Fixation: PBS + 99% ethanol (1:1; 30 min) Fluorescence microscopy	[156]
Vinegar	Acetic acid bacteria (natural)	*16S:* Komag (GAACCTTTCGGGGTTAGTG) *Fluorophore:* FITC	Bacterial removal: centrifugation (2000× *g*, 4 °C, 10 min), washed with PBS Fixation:4% paraformaldehyde (4 °C, 12 h) Flow cytometry	[157]
Glass, Polypropylene Polyethylene, Polyvinyl chloride, Copper, Silicone rubber, Stainless steel	*S. enterica*/ *L. monocytogenes/E. coli* single, dual and tri-species biofilms (laboratory grown)	*23S, PNA:* SalPNA1873 *16S, PNA:* LmPNA1253 (GACCCTTTGTACTAT) *Fluorophore:* Alexa594, Alexa488	Biofilm preparation: 24–48 h biofilm formation on different materials Sample preparation: biofilm coupons in distilled water; sonicated for 5 s at 25% amplitude; centrifugation (10,000 rpm, 5 min) Fixation: 4% paraformaldehyde for 1 h Fluorescence microscopy; confocal laser scanning microscopy	[158]
Polyvinyl chloride coupons	*H. pylori (natural)*	*PNA:* (TAATCAGCACTCTAGCAA) *Fluorophore:* carboxyfluorescein	Sampling: semi-circular flow cells containing PVC coupons placed in a bypass of a drinking water distribution system, sampling after up to 72 d Fixation: coupons in 90% ethanol, 10 min Fluorescence microscopy	[159]
Conveyor in brewery	*Natural biofilm*	EUB338 EUK502 ARCH915 ALF968 BET42a GAM42a XAN818 HGC69a LGC-354A-C CF319a PLA46 AG1427 Ent183 Pae997 Hpae1 (GAAGGCACCAATCCATC) Hpae2 (TGTCAAGGCCWGGTAAGG) *Fluorophore:* not given	Sample preparation: removal of biofilms, lubricants, and rubbed-off conveyor material sampled with sterile spatula; washing twice with sterilized water and decane; centrifugation to remove decane Fixation: paraformaldehyde or ethanol	[160]
Stainless steel coupons	*Arcobacter brutzleri* *Arcobacter cryaerophilus* *Arcobacter skirrowii* *C. jejuni* *C. coli* biofilms (spiked)	Arc94^Cy3^ (TGCGCCACTTAGCTGACA) Catherm^Cy3^ (GCCCTAAGCGTCCTTCCA) EUB338^FAM^ (GCTGCCTCCCGTAGGAGT) *Fluorophore:* Cy3, FAM	Biofilm preparation: stainless steel coupons in casein peptone soymeal-peptone broth containing 10^7^ cfu/mL bacteria; culturing for 78 h at 25 °C under aerobic or microaerobic conditions Sample preparation: coupons wiped with sterile swabs; swabs with biofilm shaken in PBS (2 min, vortex); centrifugation (16,500× *g*, 5 min, 21 °C) Fixation: 2% formaldehyde, 24 h, 4 °C Fluorescence microscopy	[161]

**Table 5:** FISH applications for microorganism detection in water samples.

Sample	Target Microorganisms	Target Probe (5′-3′ Sequence) and Fluorophore	Sample Preparation/Fixation/Observation Method	References
Water	*E. coli* (spiked)	*16S:* ES445 (CTTTACTCCCTTCCTCCC) *Fluorophore:* Cy3	Fixation: formalin (final conc. 2%) Semi-automatically polydimethylsiloxane-glass hybrid microfluidic device; fluorescence microscopy	[163]
Tap water	*C. coli* (spiked)	*16S, PNA:* CJE195 *Fluorophore:* TAMRA	Bacterial removal: samples filtered through a track etch black membrane filter (0.2 µm) Fixation: smear air dried, gently flamed; 90% ethanol (10 min), air-dried Fluorescence microscopy	[164]
Water	*Mycobacterium avium* (spiked)	*16S, PNA:* MAV148 (TGCGTCTTGAGGTCC) *Fluorophore*: 6-FAM	Bacterial removal: filtered through membrane filter (0.2 µm); filter shaking with 6 mL of the original water filtrate and glass beads Fixation: smear air dried, gently flamed; 90% ethanol (10 min), air-dried Fluorescence microscopy	[165]
Freshwater lake	*Microcystis aeruginosa* *Planktothrix rubescens* *Planktothrix agardhii* *(spiked)*	Probes labeled with horseradish peroxidase *16S:* EUB338 (GCTGCCTCCCGTAGGAGT) *16S:* MICR3 (TCTGCCAGTTTCCACCGCCTTTAGGT) *mcyA-mRNA:* MCYA (ATGAGCCGCCAATAAAACACTTT) *Fluorophore*: FITC-labeled tyramides	Sample preparation: filtration of water Fixation: 1% paraformaldehyde, 15 min, RT Fluorescence microscopy	[166]
Lakes; Oceans	Natural load	*16S:* EUB338 (GCTGCCTCCCGTAGGAGT) *16S:* NON338 (ACTCCTACGGGAGGCAGC) *16S*: ALF968 (GGTAAGGTTCTGCGCGTT) *23S:* BET42a (GCCTTCCCACTTCGTTT) *23S*: GAM42a (GCCTTCCCACATCGTTT) *16S*: CF319a (TGGTCCGTGTCTCAGTAC) *16S:* PLA886 (GCCTTGCGACCATACTCCC) 1*6S*: ARCH915 (GTGCTCCCCCGCCAATTCCT) *Fluorophore:* Cy3	Sample preparation: concentration of water samples on white polycarbonate filters (0.2 µm pore size) Fixation: 4% paraformaldehyde, 30 min, RT Fluorescence microscopy	[168]
Seawater	Natural load	*16S:* EUB338 (GCTGCCTCCCGTAGGAGT) *Fluorophore:* Cy5	Sample preparation: large plankton particles removed by gravity filtration on 10 µm mesh; gravity filtration on 3 µm polycarbonate membranes Fixation: 2% formaldehyde, 60 min, dark, RT Microfluidic flow cytometry	[167]
Seawater	*Heterosigma akashiwo* (natural)	Probes labeled with horseradish peroxidase HSIG 1451 (CCCTCGGCAAGTCACAAT) NONEUB338 (ACTCCTACGGGAGGCAGC) EUK1209 (GGGCATCACAGACCTG) *Fluorophore:* not given	Fixation: 0.1% formaldehyde, 1 h, RT Fluorescence microscopy; flow cytometry	[170]
Seawater	Marine bacteria (natural) *E. coli* (spiked)	Probes labeled with horseradish peroxidase *16S:* EUB338 *16S:* NONEUB338 *Fluorophore:* Alexa488 labeled tyramides	Sample preparation: samples pre-filtered on a 3-μm-diameter pore-size membrane Fixation: 2% paraformaldehyde, 1 h, RT Fluorescence microscopy; flow cytometry	[171]
Lake water	Ultramicrobiota (natural)	Probes labeled with horseradish peroxidase *23S:* BET42a *16S:* LD12-121 *16S:* NON338 16S: LD12-115 (CTGAACCACAAGGCAGATTCCCACAT) *Fluorophore:* Fluorescein-labeled tyramides Anti-Fluorescein-HRP conjugate	Sample preparation: samples pre-filtered on a 0.8-μm-diameter pore-size membrane Fixation: 1.7% paraformaldehyde, 15 min, 4 °C, collected on filters Fluorescence microscopy; flow cytometry	[172]
Seawater	Bacterioplankton (natural)	Probes labeled with horseradish peroxidase *23S:* BET42a (GCCTTCCCACTTCGTTT) *16S:* CF319a (TGGTCCGTGTCTCAGTAC) *16S:* EUB338 (GCTGCCTCCCGTAGGAGT) *16S:* NONEUB338 (ACTCCTACGGGAGGCAGC) *16S*: ALF968 (GGTAAGGTTCTGCGCGTT) *23S*: GAM42a (GCCTTCCCACATCGTTT) ROS537 (CAACGCTAACCCCCTCC) OM43-162 (ATGCGGCATTAGCTAACC) Nso190 (CGATCCCCTGCTTTTCTCC) Nso1225 (CGCCATTGTATTACGTGTGA) SAR86-1245 (TTAGCGTCCGTCTGTAT) *Fluorophore:* Alexa546, Alexa488	Fixation: 2% formaldehyde, <24 h, 4 °C, collected on filters Fluorescence microscopy; flow cytometry	[173]
Seawater	*E. coli* (spiked)	*16S:* Eco541 (CCGATTAACGCTTGCACC) *16S:* Eco1482 (TACGACTTCACCCCAGTC) *Fluorophore*: FITC	Sample preparation: spiked and nonspiked seawater filtered through 15 µm membrane; centrifugation (4000× *g*, 4 °C, 15 min) two times Fixation: 4% cold paraformaldehyde, 4 °C, 16–18 h Fluorescence microscopy; flow cytometry	[174]
Seawater	*Vibrio cholerae*	TaqMan probe (TCAACCGATGCGATTGCCCAAGA) *Fluorophore*: Alexa488	Fixation: 4% cold paraformaldehyde, 4 °C, overnight Fluorescence microscopy; flow cytometry	[169]

**Table 6:** FISH applications for microorganism detection in air and aerosols.

Sample	Target Microorganisms	Target Probe (5′-3′ Sequence) and Fluorophore	Sample Preparation/Fixation/Observation Method	References
Laboratory-generated bioaerosols; Native bioaerosols in swine barn	*P. aeruginosa* *E. coli* (spiked) Natural load	*16S:* TR-EUB (GCTGCCTCCCGTAGGAGT) fl-PSMg (CCTTCCTCCCAACTT) *16S:* TR-NotEUB (ACTCCTACGGGAGGCAGC *Fluorophore*: Texas Red, fluorescein	Sampling: 30 min sampling time, 12.5 L/min flow rate into 20 mL of medium or 20 L/min into 8 mL of medium Fixation: 1% formaldehyde for microscopy; 4% paraformaldehyde for flow cytometry Fluorescence microscopy; flow cytometry	[80]
Air in a sow breeding barn	Natural load	EUB mix NONEUB ALF968 ARCH915 BET42a CF319a+b CLOST I GAM42a HGC69a LGC354abc PF2 SAU SRB385 STR *Fluorophore*: FLUOS, Cy3	Sampling: filtering air onto a 25-mm-thick glass fiber filter, 1–4 d; average air flow of 200 m^3^/h; bioaerosols eluted into a sealed container by washing the filters in sterile filtered tap water Fluorescence microscopy	[175]
Bioaerosols in swine buildings	Natural load	*16S:* fl-Univ (ACGGGCGGTCGTGT(AG)C) *16S:* fl-EUB (GCTGCCTCCCGTAGGAGT) *16S:* cy-EUK (ACCAGACTTGCCCTCC) *16S:* fl-PSMg (CCTTCCTCCCAACTT) *16S:* fl-NotEUB (ACT-CCT-ACG-GGAGGCAGC) *Fluorophore*: Fluorescein, Cy3	Sampling: AGI sampler with 12.5 L/min flow rate for 40 min Fixation: 4% cold paraformaldehyde	[176]
Air from a compost plant treating	Natural load	POD-labeled probes	Sampling: MD8 air samplers with 3.0 µm gelatin filters; filters incubated on top of CASO agar (30 °C, 24–48 h) Chemiluminescence detection	[177]
Aerosols of water	*Legionella pneumophila* serogroup 1 strain (Spiked/natural)	16S: LEG705 (CTGGTGTTCCTTCCGATC) *Fluorophore*: carbocyanine	Sampling: impaction onto agar (Andersen sampler); impingement into liquid (SKC Biosampler (Arelco); filtration (collectron MD8 (Sartorius) Fixation: 3.7% formaldehyde, 30 min Fluorescence microscopy	[178]

## Nomenclature

CARD	Catalyzed reporter deposition
CTC	5-cyano-2,3-ditolyl tetrazolium chloride
cFDA	Arboxyfluorescein diacetate
DiOC_2_(3)	3,3′-Diethyloxacarbocyanine Iodide
DiBA-C_4_(3)	Bis-(1,3-Dibutylbarbituric Acid)Trimethine Oxonol
DPH	1,6-Diphenyl-1,3,5-hexatriene
DOPE	Double Labeling of Oligonucleotide Probes
ICC	Intact cell count
ELISA	Enzyme-linked immunosorbent assay
EtBr	Ethidium bromide
FCM	Flow cytometry
FISH	Fluorescence in situ hybridization
FFS	Forward scatter
FDA	Fluorescin diacetate
FACS	Fluorescence-activated cell sorters
HCR	Hybridization chain reaction
LNA	Locked nucleic acids
PNA	Peptide nucleic acids
PCR	Polymerase chain reaction
PD	Photodiodes
PMT	Photomultiplier tubes
PI	Propidium iodide
PEF	Pulsed electric fields
SSC	Side scatter
SG1	SYBR^®^ Green
TO	Thiazole orange
TCC	Total cell count
VBNC	Viable but non-culturable
